# Energetics of side-chain partitioning of β-signal residues in unassisted folding of a transmembrane β-barrel protein

**DOI:** 10.1074/jbc.M117.789446

**Published:** 2017-06-07

**Authors:** Bharat Ramasubramanian Iyer, Punit Zadafiya, Pallavi Vijay Vetal, Radhakrishnan Mahalakshmi

**Affiliations:** From the Molecular Biophysics Laboratory, Department of Biological Sciences, Indian Institute of Science Education and Research, Bhauri, Bhopal 462066, India

**Keywords:** energetics, membrane biophysics, membrane protein, outer membrane, protein folding, thermodynamics, accessible surface, hydrophobicity, membrane interface, partitioning energy

## Abstract

The free energy of water-to-interface amino acid partitioning is a major contributing factor in membrane protein folding and stability. The interface residues at the C terminus of transmembrane β-barrels form the β-signal motif required for assisted β-barrel assembly *in vivo* but are believed to be less important for β-barrel assembly *in vitro*. Here, we experimentally measured the thermodynamic contribution of all 20 amino acids at the β-signal motif to the unassisted folding of the model β-barrel protein PagP. We obtained the partitioning free energy for all 20 amino acids at the lipid-facing interface (ΔΔ*G*^0^*_w,i_*_(φ)_) and the protein-facing interface (ΔΔ*G*^0^*_w,i_*_(π)_) residues and found that hydrophobic amino acids are most favorably transferred to the lipid-facing interface, whereas charged and polar groups display the highest partitioning energy. Furthermore, the change in non-polar surface area correlated directly with the partitioning free energy for the lipid-facing residue and inversely with the protein-facing residue. We also demonstrate that the interface residues of the β-signal motif are vital for *in vitro* barrel assembly, because they exhibit a side chain–specific energetic contribution determined by the change in nonpolar accessible surface. We further establish that folding cooperativity and hydrophobic collapse are balanced at the membrane interface for optimal stability of the PagP β-barrel scaffold. We conclude that the PagP C-terminal β-signal motif influences the folding cooperativity and stability of the folded β-barrel and that the thermodynamic contributions of the lipid- and protein-facing residues in the transmembrane protein β-signal motif depend on the nature of the amino acid side chain.

## Introduction

Membrane proteins constitute one-third of the proteome of any living organism. They are vital players in important biological processes, including transport, signaling, and cell–cell communication. There is a recent interest in understanding the biogenesis, folding, and turnover of membrane proteins, particularly the transmembrane β-barrels found in the outer membrane of mitochondria, chloroplast, and Gram-negative bacteria (1–4). Collectively known as outer membrane proteins (OMPs),^4^ transmembrane β-barrels share a very simple structural framework consisting of 8–26 β-strands embedded into the membrane, arranged largely in an antiparallel fashion (5). The major contributing factor to the thermodynamic stability of OMPs is the free energy of water-to-bilayer partitioning of the lipid-facing amino acid (6). This transfer free energy from water to the bilayer midplane has been measured experimentally using an OMP for the 20 most abundant amino acids (7). This *whole-protein scale* has also been validated computationally (8). The other experimental partitioning scales derived using protein systems are available from transmembrane helices. These include the translocon scale (also considered the *biological hydrophobicity scale*), which measures the translocon-to-bilayer free energy change (9, 10), and the dsTβL system deriving insertion energetics for a single-pass transmembrane helix (11). Most of these scales report the partitioning free energy for residues to the bilayer midplane.

A reliable measure of the thermodynamics at the membrane interface is available from both experimental (11, 12) and computational methods (13). Most of our current understanding of the amino acid partitioning free energy at the interface is derived from the seminal work of Wimley and White (12). The Wimley–White scale, derived from the water-to-bilayer interface partitioning free energy of model pentapeptides, has been widely employed to describe the bilayer interface energetics. The bilayer interface is a complex environment that bridges the hydrophilic solvated exterior with the hydrophobic lipid core (14, 15). It has been argued that the unique amphiphilic chemical milieu presented at the interface demands equally complex residues in the protein structure to be positioned here (14, 16–18). Indeed, aromatic and positively charged residues are abundant at the bilayer interface, where they define the protein boundary during cotranslational folding (19), serve as post-folding protein anchors (14), and facilitate protein–protein interactions (20, 21). In Gram-negative bacteria, an additional functional importance of the interface residues at the C terminus is in OMP biogenesis.

**Figure 1.**
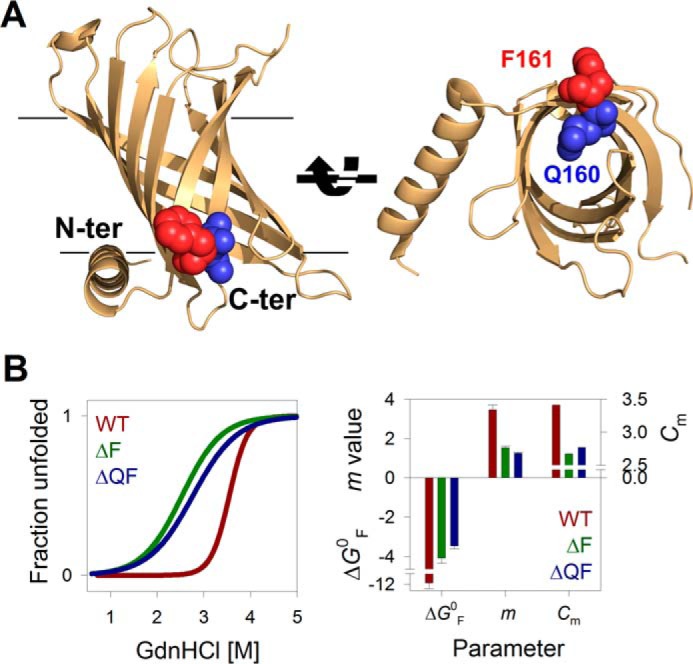
**C-terminal residues affect PagP equilibrium folding free energy.** *A*, schematic representations of *E. coli* PagP (PDB entry 3GP6) highlighting the C-terminal (Phe^161^; *maroon*) and penultimate (Gln^160^; *blue*) residues belonging to the β-signal of the 8-stranded transmembrane barrel. The structure on the *right* highlights how the Phe^161^ side chain projects toward the lipid environment, whereas that of Gln^160^ faces the protein interior. *B*, two-state equilibrium folding data of PagP-WT (*WT*; *brown*) and C-terminal deletion mutants PagP-ΔF^161^ (Δ*F*; *green*) and PagP-ΔQ^160^F^161^ (Δ*QF*; *blue*) obtained from GdnHCl-mediated chemical denaturation. Fluorescence intensities at the emission maximum were plotted as unfolded fraction from 0 to 1 and fitted to a two-state unfolding equation (fits are shown in the *left panel*; see supplemental Fig. S3 for the data) to derive the thermodynamic parameters Δ*G*^0^, *m* value, and *C_m_* (*right*). *Error bars*, goodness of fit.

The C-terminal interface residues of transmembrane β-barrels are of particular interest in OMP folding. They bear the β-signal with the consensus Aro-Xaa-Aro motif (where Aro represents an aromatic amino acid and Xaa is any amino acid) (supplemental Fig. S1) and are well conserved across all known bacterial OMPs (22, 23). The β-signal is believed to serve as the recognition sequence for OMP assembly factors such as SurA and Skp in prokaryotes (22) and their eukaryotic analogs (tiny translocases of the inner membrane (24)) during OMP biogenesis. In the model OMP PagP (PhoPQ-activated gene P), the C-terminal residues Gln^160^ and Phe^161^ form a part of the β-signal. These residues map to the interface in the folded β-barrel topology (25–27). In folded PagP, the side chain of Gln^160^ faces the barrel interior, whereas Phe^161^ points to the water-lipid interface (Fig. 1*A*). NMR measurements of PagP in *n*-dodecylphosphocholine (DPC) micelles showed that the terminal Phe^161^ represents a position of moderate hydrophobicity (28). The results from this study, which used oxygen and water contacts as probes of hydrophobicity and topology, indicate that Phe^161^ exhibits NMR characteristics that suggest an interface location, whereas the inward-facing side chain of Gln^160^ displays relaxation rates indicative of a hydrophilic region. Gln^160^ and Phe^161^ also show higher dynamicity than other strand residues (25). Additionally, Gln^160^ exhibits a high φ-value and therefore appears to be well structured in the transition state ensemble of PagP (29). Gln^160^ is also involved in hydrogen bonding with Trp^60^ in the interior of the PagP barrel and is crucial to the formation of the folded structure. Hence, Gln^160^ can be considered a part of the PagP folding nucleus (29).

Currently, there is no thermodynamic measurement available for the contribution of these β-signal residues to OMP stability, post-assembly. Here, we measured the thermodynamic contribution of all 20 residues at the β-signal motif, using the C-terminal β-signal residues of PagP. PagP is one of the few thermodynamically stabilized OMPs to exhibit reversible folding (6, 29, 30). We measured the folding free energy for all residues at the protein-facing and lipid-facing membrane interface by systematically substituting the C-terminal interface residues PagP-Q^160^ and PagP-F^161^. We found that the residue preference at the amphiphilic interface (position 161) was in favor of side-chain hydrophobicity. Further, we obtained residue dependence for β-barrel assembly from the side-chain preference at the penultimate residue (position 160). Our measured interface energetics for the β-signal residues correlates conditionally with the biological hydrophobicity scale, whereas we obtained strong correlations with the whole-protein and Wimley–White scales, with interesting deviations.

## Results

### Interface residues Gln^160^-Phe^161^ stabilize PagP through backbone hydrogen bonds

The folding of PagP *in vitro* is driven exclusively by inter- and intramolecular interactions, without assistance from accessory proteins (25, 28, 29), and would therefore not require the β-signal. To check this, we measured the thermodynamic parameters for wild-type PagP (PagP-WT) and two mutants that lacked the terminal Phe^161^ (PagP-ΔF^161^) and Gln^160^-Phe^161^ (PagP-ΔQ^160^F^161^) using equilibrium chemical denaturation measurements. We used the untagged full-length PagP overproduced as inclusion bodies in *Escherichia coli*. It is widely known that membrane proteins are rarely amenable to reversible equilibrium measurements and often exhibit hysteresis between the folding and unfolding arms (6, 31). In lipidic systems, such as 1,2-dilauroyl-*sn*-glycero-3-phosphocholine (DLPC) vesicles, PagP lacking a His_6_ tag (32) also shows hysteresis in various buffer and pH conditions. We could achieve reversible folding of untagged PagP only at pH 9.5 in DPC micelles. Other buffer systems and pH conditions (including acidic pH) showed path dependence and depressed values for folding/unfolding cooperativity. Hence, our experiments are carried out at pH 9.5. Whereas recent studies suggest that properties of membrane proteins measured *in vitro* using micellar systems, such as DPC, can represent their native behavior (33–36), whether our results from such a simplified system at non-native pH can be extrapolated to conditions *in vivo* is unclear.

The deletion of Phe^161^ is sufficient to decrease the Gibbs free energy (Δ*G*_F_^0,H2O^, indicated henceforth as Δ*G*^0^) of PagP-WT by ∼8 kcal mol^−1^ (Fig. 1*B*). This 3-fold reduction in the stability of PagP upon deletion of residue 161 suggests that phenylalanine plays an anchoring role in PagP at the membrane interface, as seen for interface aromatics in several β-barrels (37–39).

To assess whether PagP requires Phe^161^ to fold in micellar systems *in vitro*, we generated a PagP-*X*^161^ (where *X* represents any amino acid) mutant library. We found that all mutants were >85% folded, resisted proteolysis, and were enzymatically active (supplemental Fig. S1). Hence, substitution but not deletion of the C-terminal motif residue preserves the ability of PagP to fold in lipidic environments. Whereas previous studies (22, 23) have associated the Aro-Xaa-Aro motif with its role in interacting with the barrel assembly machinery, we found evidence that the C-terminal residue anchors the OMP to the membrane (Fig. 1) and facilitates barrel assembly through backbone hydrogen bonding (Fig. 1*B*).

### Thermodynamic contribution of the interface residue correlates with change in non-polar accessible surface area (ASA)

The interface residues of membrane proteins bridge the complex hydrophobic interior of the lipid bilayer with the highly polar aqueous exterior and usually have an amphiphilic nature (40, 41). The PagP-*X*^161^ library serves as an excellent model to experimentally determine the water-to-interface partitioning free energy for all 20 residues at the β-signal motif. To validate whether residue 161 is indeed located at the interface in the PagP-DPC assembly, we carried out an all-atom molecular dynamics simulation and determined the local vicinity for Phe^161^. The 5-Å vicinity of Phe^161^ shows a moderate occupancy for water, lipid headgroup, and acyl tail, which is characteristic of an interface location for this residue (for details, see supplemental Fig. S2).

**Figure 2.**
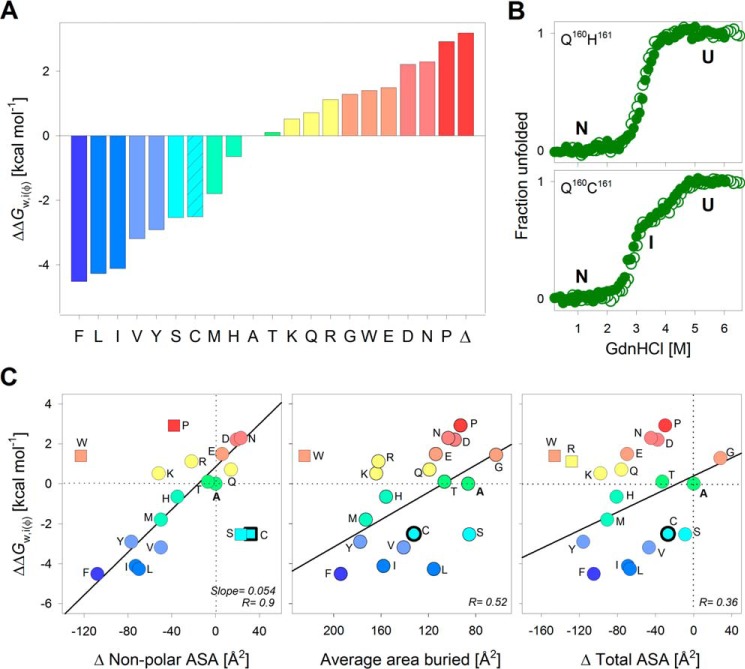
**Interface energetic contributions of the PagP terminal lipid-facing residue depends on non-polar ASA.** *A*, water-to-interface partitioning free energy calculated for all 20 residues at the lipid-facing amphiphilic position 161, normalized with respect to alanine. Amino acids are represented by their *single-letter codes*; Δ, PagP-ΔF^161^. Histograms are *colored* from *blue* to *red* in decreasing order of ΔΔ*G*^0^*_w,i_*_(φ)_. PagP-C^161^ showed a three-state folding profile (shown in *B*), and the free energy change for the more distinct transition (Δ*G*^0^_I→N_; *patterned fill* in histogram) is used here for comparison. *B*, chemical denaturation profiles exhibit no hysteresis, as seen from the overlay of folding (*open circles*) and unfolding (*filled circles*) profiles for two representative mutants. See supplemental Figs. S3 and S4 for the complete data. The Δ*G*^0^ derived from the folding titrations was used to calculate the partitioning free energies shown in *A. C*, correlations between ΔΔ*G*^0^*_w,i_*_(φ)_ and empirical parameters describing the change in ASA for each amino acid are presented as scatter plots. Linear fits to the correlation are shown as *solid lines*. Values excluded from the fits are shown as *square symbols*. Variants with three-state profiles are shown as *symbols* with *thicker edges*. The best correlation was observed with the change in non-polar ASA (*left*). Other parameters, such as average area buried upon folding (*middle*) and change in total ASA (*right*) do not correlate well with the interface partitioning free energy. The *color code* for the scatter plots is retained from *A*.

We determined the Δ*G*^0^ for each PagP mutant using equilibrium chemical denaturation measurements (Fig. 2 (*A* and *B*) and supplemental Figs. S3–S5). Except for PagP-C^161^, all of the 19 PagP-*X*^161^ constructs showed a two-state transition between the folded (F) and unfolded (U) protein states, which we fitted to a two-state linear extrapolation model (42) to obtain the Δ*G*^0^ for each mutant. From the Δ*G*^0^, we derived the energetic cost of transferring the guest amino acid at Xaa^161^ (Δ*G*^0^*_X_*) to the interface with reference to alanine (Δ*G*^0^_A_), as ΔΔ*G*^0^*_w,i_*_(φ)_ = Δ*G*^0^_A_ − Δ*G*^0^*_X_* (where φ represents the hydrophobic lipid-facing interface).

We found that the ΔΔ*G*^0^*_w,i_*_(φ)_ follows a hydrophobicity scale with hydrophobic residues at one end and charged residues at the other end, separated by alanine. The two mutants PagP-ΔF^161^ and PagP-P^161^ and ΔP^161^ are highly destabilized because they lack a backbone hydrogen bond(s). We anticipated that residues with amphiphilic or polar nature would complement the amphiphilic nature of the interface and show the highest ΔΔ*G*^0^*_w,i_*_(φ)_. Surprisingly, we obtained the highest stabilizing contribution at the interface for hydrophobic residues (Fig. 2*A*). Furthermore, aliphatic residues were marginally more stabilizing than their aromatic counterparts (Leu ∼ Phe; Ile > Tyr; Fig. 2*A*). Hence, free energy contributions at the β-signal interface show similar dependences as the hydrophobic effect seen at the bilayer midplane (7).

To identify the factors that contribute to side-chain partitioning in PagP, we correlated the ΔΔ*G*^0^*_w,i_*_(φ)_ with the ASA (43, 44) (Fig. 2*C*). We found that the ΔΔ*G*^0^*_w,i_*_(φ)_ correlated directly with the difference in non-polar buried ASA for each amino acid (*r* = 0.9; Fig. 2*C*). The slope of this linear correlation is higher (slope = 0.054) than previous reports (slope = 0.023) (12, 45) and instead resembles non-polar ASA changes for the bilayer midplane (7). This may be because our measured ΔΔ*G*^0^*_w,i_*_(φ)_ at the interface was overestimated. The dynamicity and increased hydration of the DPC micelle surface might promote further occlusion of the non-polar surface of amino acid side chains, giving rise to the overestimated ΔΔ*G*^0^*_w,i_*_(φ)_. This is a limitation in our Δ*G*^0^ measurements. Although we found that the micelle structure was largely intact within the denaturant range that we used in our experiment, the amount of DPC bound to PagP may vary. Hence, we limited our comparisons to the thermodynamics across mutants and derived the ΔΔ*G*^0^*_w,i_*_(φ)_ with respect to alanine. In this process, we assumed that the energetic contributions were normalized across the mutants for the minor artifacts arising from the use of micelles as the model system. The correlations to the average per-residue buried area and total ASA are poor (Fig. 2*C*). Hence, a major stabilizing factor at the interface residue 161 of PagP appears to be the occlusion of the non-polar region of its side chain.

We found no reorganization and restructuration in the DPC micellar system across the denaturant range used in our equilibrium folding measurements, yet we cannot rule out contributions from local structural organization in the protein-micelle system to the energetic measurements. Changes in *m* values across mutants suggest structural heterogeneity in the folded state of PagP (discussed below; see Fig. 6). Therefore, ΔΔ*G*^0^*_w,i_*_(φ)_ is a reliable, if not absolute, measure for the partitioning free energy of each residue at the interface.

Although the ΔΔ*G*^0^*_w,i_*_(φ)_ values of the majority of residues correlated well with the non-polar ASA, we obtained interesting outliers, such as Cys, Ser, and Trp, whose Δ*G*^0^ was heavily skewed. These anomalies might be due to under- and overrepresentation in the non-polar surface area calculations, respectively. The tryptophan side chain appears to be more hydrophilic than predicted, despite its bulky aromatic nature. Indeed, a detailed mutational analysis on the energetic contribution of Trp to the β-barrel OmpA revealed that the indole had a destabilizing effect at the interface (38), which is in line with our findings from PagP. Other studies have also reported a wide range of hydrophobicities for the indole side chain, from most hydrophobic (Nozaki–Tanford (46), Wimley–White octanol (45), and interface (12) scales) to moderately polar (Kyte–Doolittle (47), Eisenberg–Weiss (48), Engelman–Steiz–Goldman (49), Hessa–von Heijne (9), and Moon–Fleming (7)). Considering that the tryptophan side chain exerts an energetic barrier for incorporation at the interface (ΔΔ*G*^0^*_w,i_*_(φ)_ = 1.4 kcal mol^−1^), we surmise that this energetic cost could be offset by optimal binding to assembly factors and chaperones.

PagP-C^161^ is the only mutant that deviates from the two-state folding transitions seen for all other PagP-*X*^161^ mutants (Fig. 2*B*). We ruled out the possibility of oxidative modifications of cysteine by carrying out all experiments for these mutants under reducing environments (see gel image in supplemental Fig. S5). Further, existing hydrophobicity scales support both a hydrophilic (supported by the works of Kyte–Doolittle (47), Wolfenden (50), and Wimley–White (45) due to its polar side chain) and hydrophobic (from the Rose (51) and Janin (52) scales, due to its occurrence in hydrophobic regions in native protein structures and correlation with the non-polar ASA) nature for this residue. Such duality in the nature of cysteine, and the formation of additional interactions of the thiol with the detergent headgroup, could account for its anomalous ΔΔ*G*^0^*_w,i_*_(φ)_ at the membrane. Interestingly, the behavior of serine is analogous to that of cysteine, not threonine. Although we do not have a convincing explanation for this anomaly, we speculate that similar local interactions formed by oxygen (in Ser) and sulfur (in Cys) might give rise to the observed deviations in the Δ*G*^0^ of PagP-S^161^ and PagP-C^161^.

### Thermodynamic contribution of the penultimate interface residue to barrel folding

**Figure 3.**
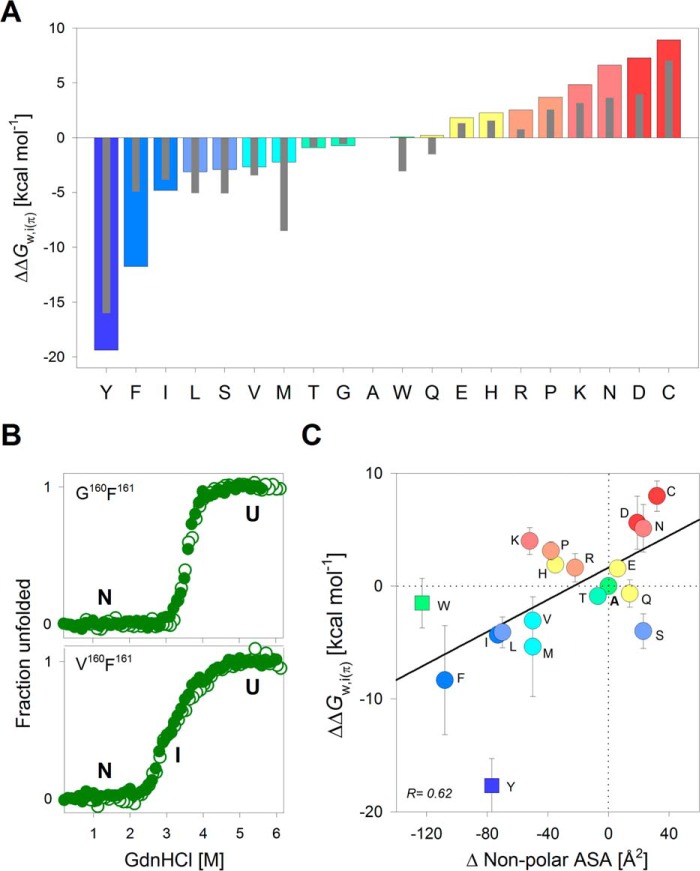
**Skewed polarity for partitioning of penultimate protein-facing interface residue of PagP.** *A*, water-to-interface partitioning free energy values calculated for all 20 residues at the protein-facing hydrophilic interface position 160, normalized with respect to alanine. *Colored bars* and *thin gray bars* represent the free energies generated from the PagP-*X*^160^F^161^ and PagP-*X*^160^L^161^ mutant libraries, respectively. Histograms are *colored* from *blue* to *red* in decreasing order of partitioning free energy values for PagP-*X*^160^F^161^. *B*, representative equilibrium chemical denaturation profiles showing overlay of folding (*open circles*) and unfolding (*filled circles*) curves for two mutants (see supplemental Figs. S3 and S6 for the complete data). *C*, correlation between ΔΔ*G*^0^*_w,i_*_(π)_ and the change in non-polar ASA is shown as a scatter plot. ΔΔ*G*^0^*_w,i_*_(π)_ values are averaged from PagP-*X*^160^F^161^ and PagP-*X*^160^L^161^ mutant libraries (*error bars* represent deviation from this average). Linear fits to the correlation are represented as *solid black lines*. Points that are excluded from the fits are shown as *square symbols*. The *color code* for the scatter plots is retained from *A*. Also see supplemental Fig. S9.

The residue Gln^160^ is located at the interface in PagP, and the side chain points to the barrel interior (Fig. 1*A*). Gln^160^ is also a likely part of the PagP folding nucleus (29). A systematic substitution at residue 160 (PagP-*X*^160^ library) would allow us to measure side-chain contributions during formation of the PagP folding nucleus. Here, we could explain the equilibrium folding transitions for some PagP-*X*^160^ mutants using a two-state model (yielding the Δ*G*^0^). However, others required a three-state model to derive the total Δ*G*^0^ of the folding process (Fig. 3 (*A* and *B*) and supplemental Figs. S3, S6, and S7). In the latter case, the Δ*G*^0^ is the sum of the change in free energy from the unfolded to the intermediate (I) state (Δ*G*_U→I_) and the intermediate to the native state (Δ*G*_I→N_).

The energetic cost of partitioning of each residue at position 160 (ΔΔ*G*^0^*_w,i_*_(π)_) with respect to alanine (ΔΔ*G*^0^*_w,i_*_(π)_ = Δ*G*^0^_A_ − Δ*G*^0^*_X_*) showed a heterogeneous distribution of polar, hydrophobic, and charged residues (Fig. 3*A*). Unlike the ΔΔ*G*^0^*_w,i_*_(φ)_ at residue 161, the ΔΔ*G*^0^*_w,i_*_(π)_ at residue 160 represents the energy required to partition an amino acid side chain from the aqueous solvent to the polar protein interior at the interface. Here, we found a skewed polarity scale, with hydrophobic residues (Tyr, Ile, and Phe) favored over hydrophilic residues. In particular, Tyr deviates considerably as it displays the highest ΔΔ*G*^0^*_w,i_*_(π)_. We speculate that the -O^ζ^H of Tyr, which adds a polar and hydrophilic nature to the phenol ring, may allow Tyr to form both polar and non-polar interactions in PagP, thereby increasing the ΔΔ*G*^0^*_w,i_*_(π)_. Further, the ΔΔ*G*^0^*_w,i_*_(π)_ is not affected by the aromatic nature of residue 161. We infer this by comparing the ΔΔ*G*^0^*_w,i_*_(π)_ derived for PagP-*X*^160^ mutants bearing an additional Phe^161^ → Leu substitution (PagP-*X*^160^L^161^ mutants) (supplemental Fig. S8). We found that the ΔΔ*G*^0^*_w,i_*_(π)_ was largely similar across all mutants, and it was only corrected for the Δ*G*^0^ ∼0.3 kcal mol^−1^ for Phe^161^ → Leu (Fig. 3*A*). Further, the ΔΔ*G*^0^*_w,i_*_(π)_ did not correlate well with the change in amino acid ASA (Fig. 3*C* and supplemental Fig. S9).

Although we found that hydrophobic residues are more stabilizing at position 160, polar residues are preferred evolutionarily. To address this, we reexamined the folding profiles. We found that when Xaa^160^ = Trp, Tyr, Phe, Leu, Ile, Val, or Met (hydrophobic residues), a stable thermodynamic intermediate was seen in the equilibrium folding profiles. We surmise that hydrophobic residues at position 160 might promote alternative folding pathways for PagP. Gln^160^ establishes multiple polar contacts in the folded PagP barrel, which can be disrupted by the formation of non-native interactions by hydrophobic residues at position 160. Due to the role of Gln^160^ in forming the folding nucleus, the PagP-*X*^160^ mutant library is well suited to provide insight into the folding mechanism of PagP. Based on our results, we speculate that the choice of the penultimate residue (Gln^160^ in PagP) may be determined by a balance between achieving a smooth folding landscape and a stable β-barrel.

An interesting observation from the ΔΔ*G*^0^*_w,i_*_(π)_ of the PagP-*X*^160^ mutants is the remarkable difference of ∼5.5 kcal mol^−1^ between Glu and Asp (or Gln and Asn). Both Glu and Asp have similar chemical properties, except for the longer side chain (additional C^γ^H_2_) in Glu and Gln. To account for this difference in ΔΔ*G*^0^*_w,i_*_(π)_, we examined the crystal structure of PagP (PDB entry 1THQ) (26). We found that Gln^160^ O^ϵ1^ and N^ϵ2^ establish weak electrostatic interactions with three spatially proximal residues, His^22^ (3.5 Å), Asp^24^ (4.7 Å), and Trp^60^ N^ϵ1^ (4.3 Å) (supplemental Fig. S10). Mutating Gln^160^ to Asp^160^ *in silico* abolishes these interactions and additionally places the carboxyl side chain of Asp^160^ within 1.7 Å of Asp^24^ (supplemental Fig. S10), giving rise to possible charge–charge repulsion. Such unfavorable interactions are less pronounced in PagP-E^160^ or PagP-Q^160^ (PagP-WT) (see supplemental Fig. S10). The destabilizing effect of Asp^160^ is also evident from the lack of a prominent gel mobility shift in the folded PagP-D^160^ protein, although the protein is resistant to proteolysis (supplemental Fig. S10). Hence, the additional C^γ^H_2_ group of Glu and Gln positions the side-chain functional groups at residue 160 away from the carboxyl group of Asp^24^, giving rise to a more favorable Δ*G*^0^ than its Asp and Asn counterparts, accounting for the measured difference in ΔΔ*G*^0^*_w,i_*_(π)_ values.

To confirm that the destabilization is indeed due to Asp^160^, we compared the results from PagP-Q^160^F^161^ and PagP-Q^160^L^161^ with two additional mutants, PagP-D^160^G^161^ and PagP-D^160^H^161^, as well as their parent constructs PagP-Q^160^G^161^ and PagP-Q^160^H^161^ (supplemental Fig. S10). This allowed us to rule out the influence of side-chain and backbone contributions as well as conformational constraints imposed by the lipid-facing residue on position 160. We found that in all cases, when Asp^160^ was present, the Δ*G*^0^ was lowered, independent of the terminal residue. These results support our conclusion that a residue-dependent contribution to the folding free energy of PagP at the penultimate position (residue 160) depends on the chemical nature and the length of the amino acid side chain. A similar destabilizing effect was seen in PagP-C^160^ (Fig. 3*A*), wherein the thiol side chain might acquire a negative charge at our experimental pH of 9.5 (p*K_a_* of the thiol group of Cys = 8.3).

### Influence of hydrophobicity of interface residue 160 on folding intermediates

Under thermodynamic equilibrium, the energetics we measure for the protein-facing PagP-*X*^160^ mutants differ in the nature of the folding transitions, because the presence of a hydrophobic residue gives rise to a prominent folding intermediate (I). Consequently, the total Δ*G*^0^_U→N_ is a sum of Δ*G*^0^_U→I_ (change in Δ*G*^0^ from U to I) and Δ*G*^0^_I→N_ (change in Δ*G*^0^ from I to N). Because the Δ*G*^0^ or free energy is a state function, the presence of the intermediate should not affect the free energy calculations. However, the nature of Xaa^160^ affects the final folded state of PagP (detailed below). Assessment of the residue contribution at the protein-facing interface (ΔΔ*G*^0^*_w,i_*_(π)_) can now vary based on whether Δ*G*^0^_U→I_ or Δ*G*^0^_I→N_ represents the true folding transition (supplemental Fig. S11). Hence, we examined the source of the rougher PagP folding landscape by comparing the folding behavior in DLPC small unilamellar vesicles (SUVs) (supplemental Fig. S12).

In membrane protein folding studies, large unilamellar vesicles (LUVs) are considered as a more reliable membrane mimetic as compared with SUVs. SUVs are not preferred due to their heterogeneity and non-equilibrium nature (53). However, we believe that this did not interfere in our measurements, because we used SUVs solely to compare the behavior of PagP across residue types. We found that full-length PagP exhibited hysteresis in both LUVs and SUVs despite prolonged incubation. The system was no longer under equilibrium, and exhibited path dependence. The measured free energy in such systems is considered an apparent value (Δ*G*^0^_app_) (54). Therefore, we derived the apparent free energy of folding (Δ*G*^0^_app,F_) for PagP in DLPC vesicles and correlated it with the Δ*G*^0^ obtained in DPC micelles.

**Figure 4.**
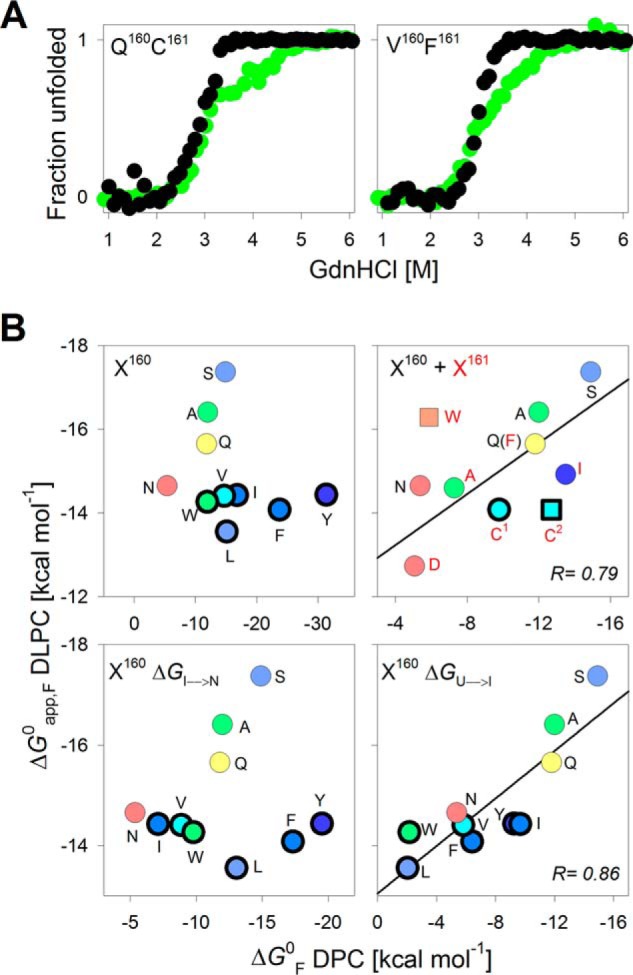
**Energetics in DLPC demarcate the two transitions observed for PagP-*X*^160^ mutants with hydrophobic substituents.** *A*, overlay of representative folding profiles for two PagP variants that exhibit three-state equilibrium folding transitions in 10 mm DPC (*green*) and the corresponding data in 0.8 mm DLPC (*black*). *B*, scatter plots correlating the Δ*G*^0^ of PagP in DPC and Δ*G*^0^_app,F_ in DLPC. The Xaa^161^ mutants (*red*) and the Xaa^160^ mutants (*black*) are represented by their *single-letter codes*. Variants with three-state profiles in DPC are shown as *symbols* with *thick edges*. Along the *x* axis, the two upper panels depict the total Δ*G*^0^ (Δ*G*^0^_U→N_ for the two-state transitions or Δ*G*^0^_U→I_ +Δ*G*^0^_I→N_ for the three-state transitions) determined for PagP-*X*^160^ mutants in DPC micelles. Along the *x* axis, the *two bottom panels* show Δ*G*^0^_I→N_ (*left*) and Δ*G*^0^_U→I_ (*right*) for the three-state transitions in DPC micelles and are presented along with the Δ*G*^0^ (DPC) for the two-state transitions. Linear fits to the correlation are represented as *solid black lines*. A good correlation (*r* = 0.79) is observed between Δ*G*^0^ (DPC) and Δ*G*^0^_app,F_ (DLPC) only for the PagP-*X*^160^ and PagP-*X*^161^ mutants showing a two-state transition (*top right*). The total Δ*G*^0^ in DPC of PagP-C^161^ is split into Δ*G*^0^_I→N_ (*C^1^*) and Δ*G*^0^_U→I_ (*C^2^*).

In DLPC, the folding profiles of all of the mutants that we tested showed a two-state transition with a global *m* value of ∼4.80 kcal mol^−1^ m^−1^, which is similar to the ∼5.4 kcal mol^−1^ m^−1^ reported for PagP-WT in DLPC LUVs (6). We also observed no detectable intermediates (Fig. 4*A*). A similar result is seen in the high detergent/protein ratio (DPR) (∼25,000:1) of DPC (supplemental Fig. S13). Hence, DLPC and high DPR of DPC allow for a concerted folding of PagP-*X*^160^ mutants (supplemental Fig. S12), and the intermediate seen when Xaa^160^ is a hydrophobic residue is due to the low DPR conditions of DPC used in this study. Because sufficient DPC molecules exist as micelles in our experimental conditions, the roughness of the PagP folding landscape and the likely formation of non-native structures seems to correlate directly with the hydrophobicity of residue 160 (supplemental Fig. S13).

We compared the total Δ*G*^0^ with the Δ*G*^0^_app,F_ obtained from DPC and DLPC, respectively, for the PagP-*X*^160^ and PagP-*X*^161^ mutants. Interestingly, despite differences in the absolute Δ*G*^0^ values, only the mutants showing a two-state transition showed an excellent linear correlation for the total Δ*G*^0^ in DPC and DLPC (Fig. 4*B*). We observed no such correlation for mutants that showed a three-state folding transition. In the latter case, we obtained a strong linear correlation between Δ*G*^0^_U→I_ and Δ*G*^0^_app,F_ (*r* = 0.86), but not between Δ*G*^0^_I→N_ and Δ*G*^0^_app,F_ (Fig. 4*B*). Similarly, the Δ*G*^0^ in high DPR of DPC was close to the Δ*G*^0^_U→I_ observed in low DPR. These results suggest that the U → I transition in DPC (Δ*G*^0^_U→I_) is a better measure of the global folding process, whereas the I → N transition (Δ*G*^0^_I→N_) might represent the formation of non-native interactions or an alternate folding pathway that is populated in mutants where Xaa^160^ is a hydrophobic residue (supplemental Fig. S13). We found that folding of PagP was hampered when a hydrophobic residue was introduced at position 160, whereas it was two-state when a polar residue was present.

The data for PagP-W^161^ and PagP-C^161^ in DLPC merit discussion. Upon comparing the DPC data for PagP-W^161^ with the Δ*G*^0^_app,F_ measured in DLPC (Fig. 4*B*), we observe that although the Trp side chain is favored at the interface in DLPC vesicles, the magnitude of stabilization does not measure up to the values presented by established interface hydrophobicity scales (10, 12). Hence, the contribution of the indole might be protein-dependent. PagP-C^161^ is the only mutant in the PagP-*X*^161^ library that exhibits a three-state folding transition. Hence, we compared the Δ*G*^0^_U→I_ and Δ*G*^0^_I→N_ in DPC for PagP-C^161^ with the Δ*G*^0^_app,F_ measured in DLPC (Fig. 4*B*). Surprisingly, we found here that the Δ*G*^0^_I→N_, but not Δ*G*^0^_U→I_, shows a good correlation with Δ*G*^0^_app,F_ (DLPC). Although this is in contrast to the correlation seen for the PagP-*X*^160^, the reason for such a deviation is currently unclear. A likely possibility is the formation of non-native hydrophobic contacts by the thiol group and lipidic micelles in the unfolded state of PagP. However, further experiments are required to validate this argument.

**Figure 5.**
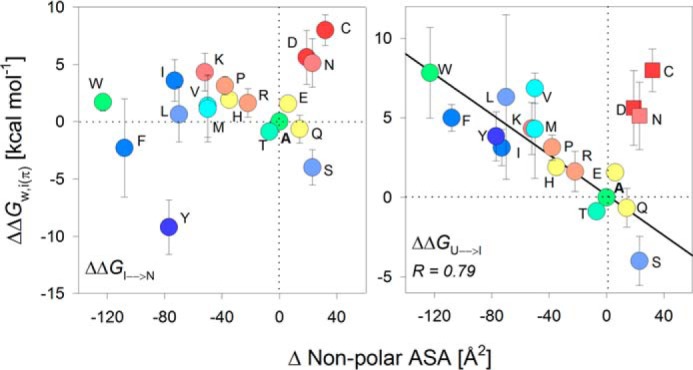
**Partitioning free energy at the protein-facing interface correlates inversely with the change in non-polar ASA of the guest residue.** Correlation plots were generated by mapping the change in non-polar ASA with the total change in folding free energy ΔΔ*G*^0^_U→N_ for the PagP-*X*^160^ mutants that exhibit two-state profiles. The plot also includes the ΔΔ*G*^0^*_w,i_*_(π)_ for the PagP-*X*^160^ mutants exhibiting three-state folding profiles (*W*, *Y*, *F*, *L*, *I*, *V*, and *M*) divided into ΔΔ*G*^0^_I→N_ (*left*) and ΔΔ*G*^0^_U→I_ (*right*). A linear (inverse) fit with a high regression coefficient (*solid line*; *r* = 0.79) could be obtained only when the ΔΔ*G*^0^_U→I_ was considered (*right*). Points that are excluded from the fits are shown as *square symbols*. The *color code* for the scatter plots is retained from Fig. 3*A*.

We also found a strong inverse correlation between the ΔΔ*G*^0^*_w,i_*_(π)_ and the non-polar ASA for each amino acid side chain when we consider Δ*G*^0^_U→N_ (two-state) and Δ*G*^0^_U→I_ (three-state) (Fig. 5). This inverse correlation was the opposite of the direct linear correlation we obtained for the lipid-facing residue (Fig. 2*C*) and is expected for the polar environment of the residue at position 160.

### Correlating partitioning free energy with atomic solvation, m value, and PagP folding landscape in micelles

The process of protein folding (*i.e.* the change from U to N state) is a highly cooperative process. This is accompanied by the change in the solvent-accessible surface area as the protein folds and is reflected in the important thermodynamic parameter termed the *m* value. By definition, the *m* value is the difference in the solvent-accessible surface area (ΔASA) between the U and N states, and it increases proportionately with the size of the protein. However, membrane proteins, including PagP, bury a larger area of their accessible surface upon folding; hence, PagP shows a higher *m* value than soluble proteins of a similar size (6, 55).

Our thermodynamic analysis reveals that PagP-*X*^160^ and PagP-*X*^161^ mutants exhibit a range of *m* values for the two-state (1–4 kcal mol^−1^ m^−1^) and three-state (0.5–8.0 kcal mol^−1^ m^−1^) folding transitions (supplemental Fig. S14). This could arise from a change in the structure of PagP upon folding or the presence of residual structure in the unfolded state. Point mutations can alter the residual structure in the unfolded protein state without affecting the folded state and thereby change the *m* value (56). In such a case, mutants with residual structure in the unfolded state have a lower *m* value, whereas the mutants without any residual structure have a higher *m* value (37, 56). A comparison of the emission profiles of the unfolded PagP mutant proteins (<λ_U_>) suggests that the unfolded protein state might not possess any significant residual structure (supplemental Fig. S15). We do not rule out the possible existence of structural heterogeneity in the unfolded state that we are unable to measure using Trp fluorescence.

**Figure 6.**
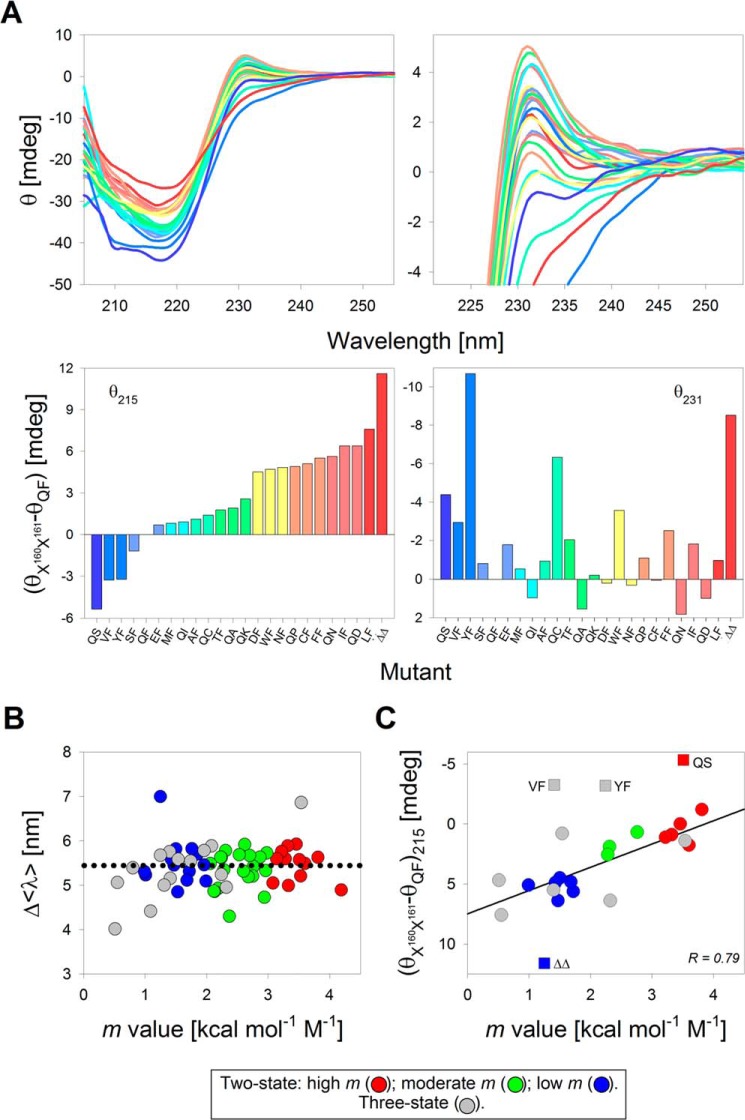
**Secondary structure content of the PagP-*X*^160^*X*^161^ mutants correlates with the measured *m* value.** *A*, far-UV CD wavelength scans recorded to examine the secondary structure content (*top left*) and tertiary aromatic interactions attained upon barrel assembly (*top right*) for a subset of PagP variants (PagP-*X*^160^ and PagP-*X*^161^) displaying a range of *m* values in DPC. The corresponding raw ellipticity values (θ, in millidegrees) at 215 and 231 nm normalized with respect to PagP-WT (*WT*) are presented as histograms in the *bottom panels*. The mutants are arranged and *color-coded* in decreasing order of θ_215_, from *dark blue* (highest θ_215_) to *dark red* (lowest θ_215_) in the *two left panels*. This *color scheme* is retained in the *right panels*. Mutants are named with *single-letter codes* for residues present at the terminal two positions; PagP-ΔQ^160^F^161^ is depicted as ΔΔ. *B*, correlation plot between changes in the Δ〈λ〉 (= 〈λ_U_〉 − 〈λ_F_〉) between the folded and unfolded states of PagP-*X*^160^ and PagP-*X*^161^ variants and the measured *m* value in DPC. The scatter plot is *colored* based on the *m* value of the mutants, with the two-state mutants divided into high (*red circles*), moderate (*green circles*), and low (*blue circles*) *m* values and the *m*_U→I_ for the three-state mutants represented as *gray circles. C*, correlation plot between the change in θ_215_ and the *m* value for all of the mutants shown in *A* (also see supplemental Fig. S16). The color code is retained from *B*. A linear fit to the correlation is shown as a *solid black line* (*r* = 0.79). Points excluded from the fit are shown as *square symbols*.

Interestingly, far-UV and near-UV CD structural analysis at 215 nm (θ_215_) and 231 nm (θ_231_) (57) show that the increase in the secondary structure content (θ_215_) of folded PagP can account for the observed increase in *m* values (Fig. 6 and supplemental Fig. S16). Comparable values of tryptophan lifetimes (average value of ∼3.39 ns) and anisotropy (∼0.098) obtained for ∼20 representative mutants in their folded state (supplemental Table S2) support the formation of a well-structured PagP β-barrel scaffold. Hence, the difference in the *m* value appears to be a function of the secondary structure attained in the folded state (Fig. 6*C*). Additionally, the *m* value shows a strong linear correlation with the difference in non-polar ASA of each lipid- or protein-facing residue (supplemental Fig. S17). The *m* value is expected to change under conditions of hydrophobic mismatch between the transmembrane region and the lipid bilayer (58). However, DPC micelles impose little or no lateral pressure during PagP folding and are highly dynamic entities when compared with DLPC. Hence, DPC might allow structural heterogeneity in the folded state of mutants, thereby altering the *m* value in a residue-dependent manner.

A quantitative indication of the energetics involved in the partitioning process between two phases is provided by the atomic solvation parameter (ASP). At the water-lipid interface, the ASP value that we derive for the insertion of hydrophobic residues is 0.043 ± 0.01 kcal mol^−1^ Å^−2^ (*r* = 0.90) (Fig. 2*C*), which is >3 times higher than the ASP of ∼0.0131 kcal mol^−1^ Å^−2^ for the Wimley–White interface scale (12) but closer to the ∼0.023 kcal mol^−1^ Å^−2^ obtained for folding of the OmpLA β-barrel into phosphocholine vesicles (7). The abnormally large value of ASP may arise from ASA (*m* value) changes between the folded and unfolded states of PagP mutants in DPC micelles. However, we obtain a strong linear correlation (Fig. 4*B*, *r* = 0.79, slope = 0.31) for our data from DPC micelles and DLPC vesicles. Moreover, DPC reorganization and local structural heterogeneity do not influence our measurements. Therefore, the structural variability of the folded state, which appears to be the major drawback of the PagP-DPC system, prevents quantitative assessment of side-chain ΔΔ*G*^0^ but provides a reliable qualitative readout for interface energetics.

The high value of ASP that we observed might also be explained as follows. When we compared the free energy values for PagP-WT in DPC micelles and DLPC SUVs, we observed a difference of ∼4 kcal mol^−1^ (see Fig. 4*B*). The process of folding in vesicles yielded a higher free energy value. This could be due to one of two reasons: (i) folded PagP establishes better protein–lipid contacts in DLPC vesicles than in DPC micelles due to matching hydrophobic thickness, or (ii) the native state is identical in both systems, but the denatured state ensemble is not completely unfolded in both cases. By considering either possibility, we can conclude that in DLPC vesicles, we are able to capture the thermodynamic transition of a higher magnitude. And yet, when we correlate the Δ*G*^0^ for DPC micelles with that of the DLPC SUVs, it is the micellar reaction that exhibits the higher magnitude (slope = 0.31 for DLPC *versus* DPC) (Fig. 4*B*). This would imply that the hydrophobic contribution per residue is magnified in the case of DPC micelles. Therefore, what works as an inhibitory factor in the computation of absolute free energy values (*e.g.* dynamicity of DPC micelles and the occurrence of a rougher folding landscape that probably promotes non-native interactions) proves to be an amplifying factor when the side-chain hydrophobicity is measured.

We found that the physico-chemical property of the residue 160 side chain regulates the β-barrel thermodynamic free energy landscape. Gln^160^ is a part of the PagP folding nucleus (29); therefore, side-chain partitioning energetics derived from the PagP-*X*^160^ library must be interpreted with caution. Here, we found that polar side chains are preferred over large bulky groups, because the latter influences the PagP folding landscape by stabilizing folding intermediates. On the other hand, the selection of Phe^161^ has previously been attributed as important for both its hydrophobicity and aromaticity (59). However, we found that Tyr and Trp are less stabilizing than Phe, whereas aliphatic hydrophobic residues, such as Ile, Val, and Leu, are preferred. Hence, we surmise that the hydrophobic contribution of Phe^161^ is more pronounced than its aromaticity. Furthermore, extrapolating the linear correlation between DPC and DLPC provides us with an energy contribution of ∼2 kcal/mol for the interface hydrogen bond in DLPC. In comparison, an interface hydrogen bond contributes ∼0.6 kcal mol^−1^ to the stability of transmembrane helices (60). Hence, the contribution of hydrogen bonding appears to be significant for PagP folding, especially when we consider that strand assembly and barrel closure in PagP is driven by interstrand hydrogen bonding.

### Correlating side-chain transfer free energy for β-signal residues with reported scales

**Figure 7.**
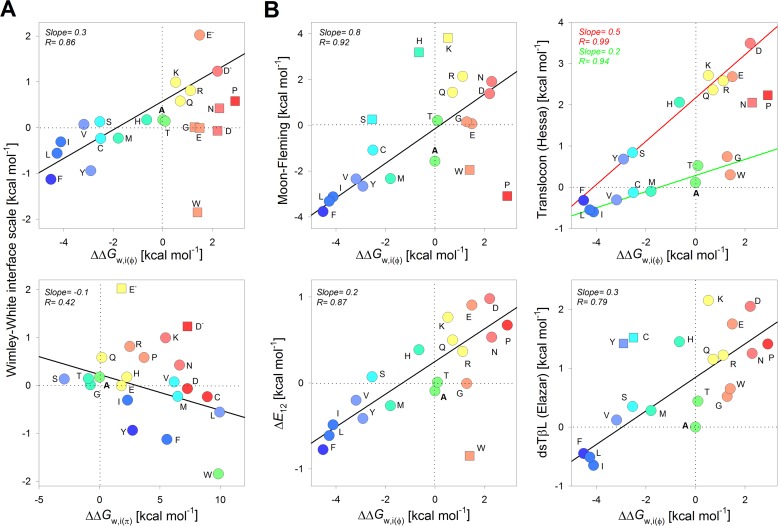
**Comparison of interface energetics for PagP β-signal residues with previously reported scales.** *A*, correlation plots generated by comparing the ΔΔ*G*^0^*_w,i_*_(φ)_ (derived from the lipid-facing interface position; *top*) and the ΔΔ*G*^0^*_w,i_*_(π)_ transfer free energy (derived from the polar protein-facing interface position; *bottom*) with the Wimley–White interface scale (12). Here, the ΔΔ*G*^0^*_w,i_*_(π)_ scale is derived from the ΔΔ*G*^0^_U→I_ (*W*, *Y*, *F*, *L*, *I*, *V*, and *M*) or ΔΔ*G*_U→N_ observed at the penultimate residue. *B*, comparison of ΔΔ*G*^0^*_w,i_*_(φ)_ with the whole-protein (7), translocon (9), Δ*E*_12_ (13), and dsTβL (11) scales. Linear fits to the correlation are represented as *solid lines*. Points excluded from the fits are shown as *square symbols. Color codes* for residues are retained from Fig. 2*A* for ΔΔ*G*^0^*_w,i_*_(φ)_ and Fig. 3*A* for ΔΔ*G*^0^*_w,i_*_(π)_. The slope and corresponding regression coefficients are indicated within each *panel* and are *color-coded* for the fits to the translocon scale in *B*.

A major driving factor in membrane protein folding is the partitioning of hydrophobic residues from the aqueous milieu into the lipid bilayer. Not surprisingly, a preferential distribution of residues was observed in membrane proteins, as the structure traverses the bilayer (14, 18). Several theoretical and experimental studies have correlated the hydrophobicity of an amino acid side chain with the free energy of its transfer from water to an apolar environment (7, 9, 11, 12, 45, 47). Hence, we asked whether the ΔΔ*G*^0^*_w,i_* that we measured for each residue at the β-signal correlates with established hydrophobicity scales (Fig. 7 and supplemental Figs. S18 and S19). Although our thermodynamic measurements with the PagP-*X*^161^ interface mutants were in DPC micelles (Fig. 2*A*), and results from such a simplified system cannot be directly extrapolated to conditions *in vivo*, considering the limited information available for the interface, a comparison of our ΔΔ*G*^0^*_w,i_* values with existing scales might provide interesting insights.

For the lipid-facing interface (ΔΔ*G*^0^*_w,i_*_(φ)_; Fig. 7), fits of the correlation plots with the Wimley–White interface scale (12) gave us a slope of 0.3 (*r* = 0.86), the Moon-Fleming scale (7) gave a slope of 0.8 (*r* = 0.92), the *E_z_* potential (13) at 12 Å from the midplane (as an indicator of interface position) gave a slope of 0.2 (*r* = 0.87), and the dsTβL scale (11) gave a slope of 0.3 (*r* = 0.79). Although we observe good correlations for the ΔΔ*G*^0^*_w,i_*_(φ)_ with all of the scales, the low value of slope of ≤0.3 indicates that the energetic cost of partitioning is overestimated in our measurements. Moreover, the structural heterogeneity of the folded state (Fig. 6*C*) and associated changes in ASA pose major limitations to our current analysis. The Wimley–White interface scale includes the contribution of the peptide bond in the partitioning free energy calculations and might account for the low value of slope. Nevertheless, one similarity in the correlations is that the positively charged residues are more favorable at the water-bilayer interface compared with negatively charged residues.

The high slope (0.8) and correlation (*r* = 0.92) for the ΔΔ*G*^0^*_w,i_*_(φ)_ with the Moon-Fleming scale (Fig. 7*B*) might suggest that the lipid-facing β-signal residue of PagP in DPC favors hydrophobicity for partitioning at the interface. Most known scales use transmembrane helices as their model systems for partitioning free energy measurements (note how R is high for the correlation with both the Δ*E*_12_ and dsTβL scales in Fig. 7*B*, whereas the slope is low). Unlike helical proteins, β-sheet structures occlude more non-polar surface area (61). The Moon–Fleming scale uses a β-barrel protein, which could also account for why we obtain the higher slope of 0.8 (Fig. 7*B*). The noticeable deviation is for partitioning of charged residues; whereas the Moon–Fleming scale assigns a higher energy penalty at bilayer midplane, it appears to be more energetically favorable at the interface (Fig. 7*B*, *top left*).

The hydrophobicity of tryptophan exhibits a noticeable difference in most of the correlations. Whereas all scales (except dsTβL) assign an energetic preference for tryptophan at the interface due to its aromaticity and amphiphilic nature, our measurements seem to account for only the hydrophobicity of the indole (Fig. 7). As a result, the partitioning of Phe and Tyr at the interface are more favorable than that of Trp. Upon correlation with the depth-dependent hydrophobicity scale, the *E_z_* potential (13), we observe that Trp is an outlier for both the midplane and interface positions (Fig. 7*B* and supplemental Figs. S19 and S20). This deviation is not surprising, because studies using OmpA have shown that Trp exhibits dual behavior (hydrophobicity and amphiphilic nature) in membrane proteins (37). Similarly, PagP-W^161^ exhibits a destabilizing effect, because Trp appears to be more hydrophilic than its calculated non-polar ASA at the interface. Electrostatic interactions of the indole with DPC may explain the reduced hydrophobicity of the indole. The contribution of an interface Trp to membrane protein stability may depend on its local environment and, therefore, be protein-specific. Such a protein-specific contribution could explain why the ΔΔ*G*^0^*_w,i_*_(φ)_ that we measure for Trp correlates poorly with established scales (Fig. 7).

The ΔΔ*G*^0^*_w,i_*_(φ)_ also correlates conditionally with the translocon scale (9), wherein hydrophobic residues (Ile, Phe, Leu, Val, Cys, Met, Ala, Trp, and Gly) correlate with a slope of 0.2 (*r* = 0.94), and hydrophilic residues (Ser, Gln, His, Leu, Arg, Glu, Asp, and Tyr) fit to the linear regression with a slope of 0.5 (*r* = 0.99) (Fig. 7*B*). Notably, Tyr correlates with other hydrophilic residues. Such a conditional correlation brings out an interesting insight into our current understanding of membrane energetics. It is believed that the magnitude of the partitioning free energy in the translocon scale is underestimated (62), because it is believed to represent the side-chain transfer free energy from the interior of the translocon to the interior of the membrane instead of transfer from water to the membrane. As a result, the translocon scale also shows a depressed value for the ASP (62). This effect is now evident when we compare only the hydrophobic residues (Fig. 7, *top right*, *green line*), wherein we observe a slope of 0.2, as opposed to the correlation seen with hydrophilic residues (slope = 0.5). Such a conditional correlation highlights the difference in partitioning energy of hydrophobic and hydrophilic residues into the lipid membrane.

For the protein-facing interface (ΔΔ*G*^0^*_w,i_*_(π)_; Fig. 7*A*, *bottom*), the correlation shows a negative slope and a poor *r* value, affirming the anticipated inverse relationship between the partitioning free energy at the polar interface, ΔASA (non-polar) (see Fig. 5), and reported hydrophobicity scales. The main reason for this seemingly anomalous observation is that the protein-facing polar interface prefers small and polar side chains over charged amino acid residues due to steric constraints during scaffold packing (see supplemental Fig. S10). Further, charged residues, such as Arg and Lys, are placed closer to hydrophobic residues (note how ΔASA for Arg and Lys is higher than Ala in Fig. 5 (*right*)) in the non-polar ASA calculations than in conventional hydrophobicity scales, mainly due to the aliphatic segments in their side chains. Hence, our ΔASA calculation (Fig. 5) and ΔΔ*G*^0^*_w,i_*_(π)_ reflect the tendency of the non-polar region of side chain to occlude itself from water.

## Discussion

The folding of the nascent OMP polypeptide is widely accepted to be a multistep process (63–65). Physico-chemical properties of the lipid (66), chaperone-dependent factors (67), and the energetic contribution of the protein primary sequence (6, 68) together play an important role in OMP assembly. The bilayer interface is amphipathic (zwitterionic), whereas the midplane is hydrophobic (apolar), yet we found that the side-chain partition energetics that we measured for the interface residues correlates surprisingly well to free energy measurements at the bilayer midplane (Moon–Fleming scale (7); Fig. 7*B*) and shows a matching magnitude. Hence, the energetic contribution for each residue at the β-signal might have considerable contributions from hydrophobic forces and change in non-polar ASA. The correlation that we observe with other reported biological hydrophobicity scales (Fig. 7 and supplemental Figs. S18 and S19) also indicates that physico-chemical factors that determine the partitioning free energy at the bilayer midplane (for both α-helical and β-barrel structures) and the interface might indeed be similar and differ only in the magnitude of change in the measured free energy values (*e.g.* see supplemental Fig. S20). At the same time, the interesting deviations that we and others (38, 45, 47, 51) observe for specific residues, such as tryptophan and cysteine, might reflect a more protein-specific contribution in transmembrane β-barrels. For example, the residue preference at the terminal position, Phe over Trp (supplemental Fig. S1*A*), may be due to the higher stability provided by the Phe side chain.

We found that the mere hydrophobicity of an amino acid side chain at the β-signal motif is energetically sufficient to stabilize the barrel scaffold. Hence, we surmise that specific *in vivo* stop transfer signals (14) (Trp, Arg, Lys, etc.) should bear greater functional relevance than serving as passive markers for membrane protein translocation. Furthermore, in accordance with Anfinsen's hypothesis (69), the asymmetric distribution of hydrophobic side chains at lipid-facing sites, polar residues at the extramembranous regions, and intra- and interprotein contact sites provides a sufficient molecular footprint to fold the nascent polypeptide. The interiors of many OMP barrels are enriched with polar residues that pose a high-energy barrier (7) when partitioned in the hydrophobic bilayer interior. Strong hydrophilic interactions established between charged and polar residues (as seen in transmembrane helices in the membrane (7, 70, 71)), along with interstrand backbone hydrogen bonds, might together contribute to the unusually high stability of OMPs (6).

Our study shows that the PagP folding pathway is strongly modulated by the physical properties of the lipidic environment, with higher lateral bilayer pressure promoting a smoother folding landscape. Similar observations in other model OMPs (58) suggest that *in vivo*, the lateral bilayer pressure works in concert with the barrel assembly machinery (BAM) (3, 67) complex to drive OMP folding. The BAM complex, lipid headgroup, and lateral bilayer pressure may be important to suppress off-pathway kinetic intermediates (66, 72, 73) or alternate folded structures (as seen in some of our PagP mutants). This argument also explains why the specific choice of residues at the C-terminal Aro-Xaa-Aro motif is important for unassisted OMP folding *in vitro*.

In conclusion, we find not only that the PagP C-terminal β-signal motif is a recognition signal for the Skp–BAM complex (22) but that it can considerably influence the folding cooperativity and stability of the folded β-barrel. The thermodynamic contribution of the lipid- and protein-facing interface residues that form a part of the crucial β-signal motif of transmembrane OMPs depends considerably on the nature of the amino acid side chain. Overall, the interface partitioning energetics that we have determined for PagP will instigate further measurements of a whole-protein interface scale for transmembrane proteins in near-native environments.

## Experimental procedures

### Molecular dynamics simulations

All molecular dynamics simulations were performed at a constant temperature of 310 K and a constant pressure of 1 bar, using GROMACS version 5.0.4 (74), using previously reported protocols (30). Using the CHARMM-GUI (75–77) input generator, we embedded the protein molecule (PagP crystal structure, PDB code 1THQ (26)) in a micelle system consisting of 80 DPC molecules. We first equilibrated the protein–micelle complex and followed it up with a production molecular dynamics simulation of 10 ns. Vicinity analysis was performed using VMD (Visual Molecular Dynamics) (78).

### Protein preparation

The wild-type *pagP* gene (accession number NC_000913) was amplified from the *E. coli* K12 MG1655 genome using specific primers (supplemental Table S1) and cloned without the signal sequence into pET-3a vector between NdeI and BamHI sites (30). This was used as the template to generate a library of 60 mutants at the C-terminal residues 160 and 161, which included Gln^160^-Xaa^161^, Xaa^160^-Phe^161^, and Xaa^160^-Leu^161^ (where Xaa is any one of 20 naturally occurring amino acids). Further, the residue deletion mutants ΔF^161^ and ΔQ^160^F^161^ were also generated. PagP-WT and all of the mutants were expressed without any affinity tags. *E. coli* BL21(DE3) cells were transformed with these plasmids and were used for producing the protein as inclusion bodies. The proteins were processed further to ∼95% purity using reported methods (79).

### PagP folding in DPC

For the equilibrium folding and unfolding measurements of PagP, stock protein was generated using reported methods (29, 30). Briefly, the unfolded protein was dissolved in 20 mm Tris–HCl, pH 9.5, containing 8 m urea, at a concentration of ∼300 μm. This was diluted 10-fold into the folding reaction containing 100 mm DPC prepared in 20 mm Tris–HCl, pH 9.5, at 4 °C. The sample was heated at 70 °C for 3 min (80), immediately transferred to 4 °C, and incubated overnight. The next day, samples were centrifuged at 16,600 × *g* for 1 h to remove any trace amounts of protein aggregates. Each preparation was checked for soluble aggregates on a UV spectrophotometer by monitoring the scattering between 300 and 340 nm and electrophoretically using cold SDS-PAGE (66, 80, 81). The final stock contained ∼30 μm PagP in 100 mm DPC and 20 mm Tris–HCl, pH 9.5. This corresponds to a DPR of ∼3300:1. The folding efficiency for this stock was quantified using densitometry analysis of the gel mobility shift (66, 80, 81) and proteolysis by proteinase K (30). The unfolded stock was prepared similarly, with the only difference that all the solutions contained 8 m GdnHCl.

### PagP folding in DLPC

PagP exhibits hysteresis in all of the conditions of protein-lipid ratios, buffer, pH, and temperature that we were able to screen. Hence, we studied only the folding equilibrium of PagP in SUVs of DLPC, prepared by sonication. For the folding measurements of PagP, unfolded protein (2.6 mm PagP in 7 m GdnHCl prepared in Tris–HCl, pH 9.5) was added in a 1:9 ratio to 89 mm DLPC SUVs. This mixture was further 10-fold diluted in 7 m GdnHCl to yield the folding stock containing 26 μm protein and 8 mm lipid. This stock was 10-fold diluted into varying GdnHCl concentrations ranging from 0.7 to 7 m, and these final reactions were used for equilibrium fluorescence measurements.

### Equilibrium (un)folding experiments using steady-state fluorescence

The PagP samples for equilibrium folding and unfolding in DPC were prepared by 10-fold dilution of the respective protein stocks in various GdnHCl concentrations ranging from ∼0.7 to ∼6.6 m, at an increment of 0.1 m. This gave us a final protein concentration of 3 μm and DPC of 10 mm in each reaction. Samples were incubated at 25 °C, and fluorescence measurements were acquired on a microplate reader at the same temperature. We monitored the progress of the reactions using the decrease in tryptophan fluorescence emission intensity, with increase in GdnHCl concentration. Spectra were acquired using a λ_ex_ of 295 nm and λ_em_ of 320–400 nm. For PagP and its mutants, equilibrium for the reaction was achieved within 24 h.

From the fluorescence profiles, we calculated the unfolded fraction (*f*_U_) for the 48-h data using the following equation.

(Eq. 1) $$f_{\text{U}}=\frac{y_{\text{O}}-\left(y_{\text{F}}+m_{\text{F}}\left[D\right]\right)}{\left(y_{\text{U}}+m_{\text{U}}\left[D\right]\right)-\left(y_{\text{F}}+m_{\text{F}}\left[D\right]\right)}$$

Here, *y*_O_ is the observed fluorescence at GdnHCl concentration [D], whereas *y*_F_, *m*_F_, *y*_U_, and *m*_U_ are intercepts and slopes of the pre- and post-transition baselines, respectively.

We were able to explain the folding transitions for most of the mutants using the two-state equation (42).

(Eq. 2) $$f_{\text{U}}=\frac{\exp\left(-\left(\Delta G+m\left[D\right]\right)/RT\right)}{1+\exp\left[-\left(\Delta G+m\left[D\right]\right)/RT\right]}$$

This equation assumes that the protein folds in a cooperative manner from the unfolded (U) to the folded (F) state, without a detectable folding intermediate. We obtained the thermodynamic parameters Δ*G*^0^ (Δ*G*_F_^0,H2O^, folding free energy) and *m* value (change in ASA between U and F states) of folding from the fits. The midpoint of chemical denaturation (*C_m_*) was calculated as *C_m_* = Δ*G*/*m*.

The folding transition of some mutants could only be explained using a three-state equation (Equation 3), due to the occurrence of an intermediate (I) (7).

(Eq. 3) $$f_{\text{U}}=\frac{\left(y_{\text{F}}+m_{\text{F}}\left[D\right]\right)+\left(\exp\left(-\frac{\Delta G_{1}+m_{1}\left[D\right]}{RT}\right)\left(y_{\text{I}}+m_{\text{I}}\left[D\right]\right)\right)+\left(\exp\left(-\frac{\Delta G_{1}+m_{1}\left[D\right]}{RT}\right)\exp\left(-\frac{\Delta G_{2}+m_{2}\left[D\right]}{RT}\right)\left(y_{\text{U}}+m_{\text{U}}\left[D\right]\right)\right)}{1+\exp\left(-\frac{\Delta G_{1}+m_{1}\left[D\right]}{RT}\right)+\exp\left(-\frac{\Delta G_{1}+m_{1}\left[D\right]}{RT}\right)\exp\left(-\frac{\Delta G_{2}+m_{2}\left[D\right]}{RT}\right)}$$

Here, we obtained Δ*G*_1_ and Δ*G*_2_ and their corresponding *m*_1_ and *m*_2_ values for the change in free energy from the first (U → I) and second (I → N) transitions, respectively.

The PagP samples for folding in DLPC were prepared as described above. We used the same parameters for data acquisition in DLPC samples as for DPC. From the fluorescence profiles, we calculated the unfolded fraction (*f*_U_) for the 48-h data using Equation 1. The data were fitted globally to Equation 2, assuming a common *m* value. Fits of the data to Equation 2 yielded the apparent thermodynamic parameters, namely the apparent change in free energy Δ*G*_app_, apparent ASA change *m*_app_, and *C_m_* for each mutant.

### Fluorescence anisotropy, lifetime, and average emission wavelength measurements

For PagP and its mutants, equilibrium for the chemical denaturation was achieved within 24 h. Tryptophan fluorescence anisotropy and lifetimes were measured 24–48 h post-equilibrium. We used a λ_ex_ of 295 nm. Anisotropy was monitored at 344 nm (λ_em-max_ of folded PagP) and lifetime at 340 nm. The instrument parameters reported previously (30) were followed here. We fitted the decay curves for lifetime measurements to a triple exponential decay function and calculated the average lifetime as 〈τ〉 = Σ(τ_i_·α*_i_*).

Average emission wavelength (〈λ〉) was calculated from the emission spectra using Equation 4, as follows (82).

(Eq. 4) $$\lambda =\sum (I\left(\lambda_{i}\right)\lambda_{i}/\sum \left(I\left(\lambda_{i}\right)\right)$$

Here, λ*_i_* is the *i*th emission wavelength, and *I*(λ*_i_*)is the emission intensity at that wavelength. The data were further corrected using a normalization factor (*Q*_R_) so that the 〈λ〉 is now linearly proportional to the unfolded protein fraction in solution (82).

### Enzymatic assay

We monitored the activity of PagP refolded in DPC micelles by measuring the release of *p*-nitrophenol through the hydrolysis of *p*NPP (30). Briefly, we added 0.04 μg/μl (2 μm) refolded protein to 1 mm of the substrate analogue *p*NPP prepared in 2% Triton X-100. We monitored the release of *p*-nitrophenol spectrophotometrically at 405 nm. The rate of the reaction was monitored for 60 min, following which we used the linear region of the kinetics curve for deriving PagP activity (30).

### Circular dichroism measurements

We recorded the far-UV CD wavelength scans of 0.3 μg/μl protein in 50 mm DPC using a 1-mm quartz cuvette, between 205 and 260 nm at a temperature of 5 °C, as described previously (30). Data were averaged over three accumulations, corrected for buffer contribution, smoothed, and represented as molar ellipticity (degrees cm^2^ dmol^−1^) values calculated using reported methods (80).

## Author contributions

R. M. conceived, designed, and coordinated the study and wrote the paper. B. R. I. performed the experiments shown in Figs. 1, 2, 3, 5, and 7 and contributed to writing the paper. P. Z. performed the experiments shown in Figs. 3–7 and contributed to writing the paper. P. V. V. performed the experiments shown in Figs. 2 and 4. All authors analyzed the results and approved the final version of the manuscript.

## Supplementary Material

Supplemental Data

## Footnotes

4 The abbreviations used are:

OMP: outer membrane protein
ASA: accessible surface area
ASP: atomic solvation parameter
BAM: β-barrel assembly machinery
*C_m_*: chemical denaturation midpoint
DLPC: 12:0 PC, 1,2-dilauroyl-*sn*-glycero-3-phosphocholine
DPC: *n*-dodecyl phosphocholine
DPR: detergent/protein ratio
*f*_U_: unfolded fraction
Δ*G*^0^: Gibbs free energy of folding
GdnHCl: guanidine hydrochloride
LUV: large unilamellar vesicle
*m*: folding cooperativity
*p*NPP: *p*-nitrophenyl palmitate
SUV: small unilamellar vesicle
Aro: aromatic residue
PDB: Protein Data Bank.
