# Supplementary material for: Energetics of side-chain partitioning of β-signal residues in unassisted folding of a transmembrane β-barrel protein
Source: J Biol Chem. 2017 Jun 7;292(29):12351–65. doi: 10.1074/jbc.M117.789446 (PMC5519381; doi:10.1074/jbc.M117.789446)
Supplement: Supplemental Data [file supp_292_29_12351__index.html]

Energetics of side chain partitioning of β-signal residues in unassisted folding of a transmembrane β-barrel protein — Energetics of side-chain partitioning of β-signal residues in unassisted folding of a transmembrane β-barrel protein — Side-chain interface energetics in PagP folding — Supplemental Data 

# Energetics of side-chain partitioning of β-signal residues in unassisted folding of a transmembrane β-barrel protein

## Supplemental Data

- Supplemental Information (.pdf, 3.6 MB) - Supplemental Figures and Tables.
