# Supplementary material for: Energetics of side-chain partitioning of β-signal residues in unassisted folding of a transmembrane β-barrel protein
Source: J Biol Chem. 2017 Jun 7;292(29):12351–65. doi: 10.1074/jbc.M117.789446 (PMC5519381; doi:10.1074/jbc.M117.789446)
Supplement: Supplemental Data [file 10.1074_M117.789446_jbc.M117.789446-1.pdf]

Energetics of side shain partitioning of β-signal residues in unassisted folding of a transmembrane β-barrel protein

Bharat Ramasubramanian Iyer,[‡] Punit Zadafiya,[‡] Pallavi Vijay Vetal, and Radhakrishnan Mahalakshmi.*

Molecular Biophysics Laboratory, Department of Biological Sciences, Indian Institute of Science Education and Research, India.

[‡]Equal contribution. *Corresponding author. E-mail: maha@iiserb.ac.in

SUPPLEMENTAL INFORMATION

Supplemental Figures

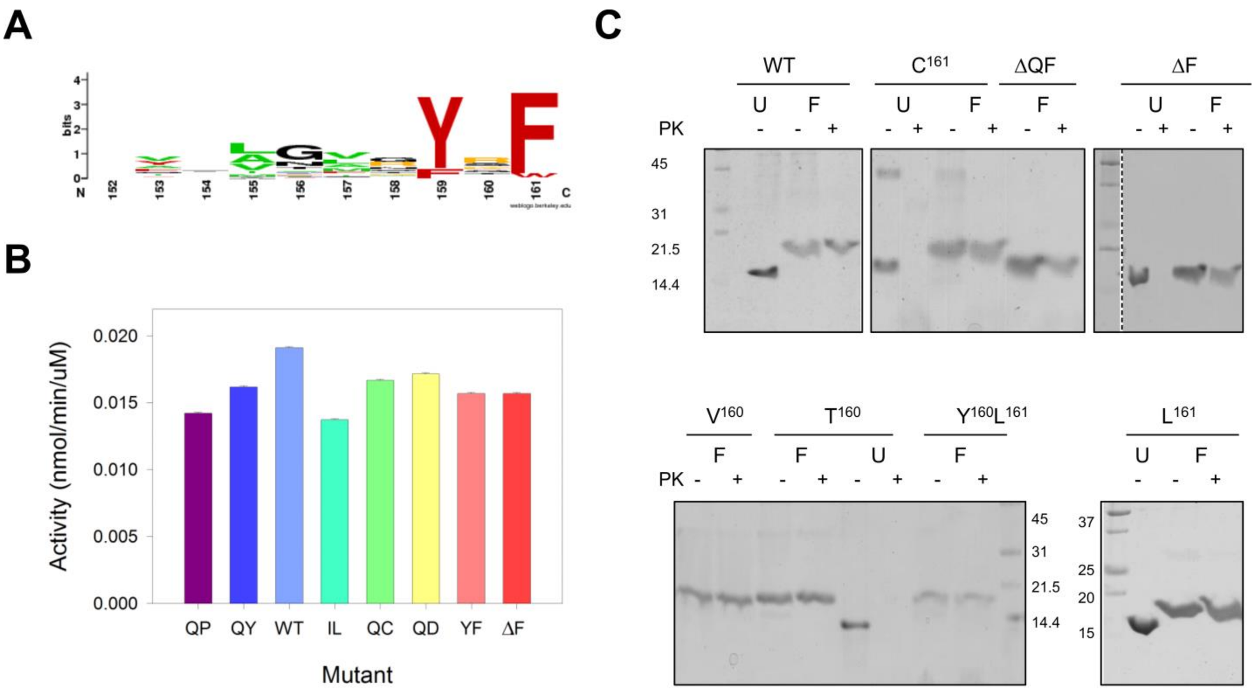

Figure S1. Folding of PagP variants monitored by phospholipase hydrolysis measurements and gel shift assay. (A) Multiple sequence alignment of the β-signal from 31 OMPs from several Gram-negative bacteria, presented in WebLogo (1) format. The residue numbering for PagP is retained here, and is presented in the N→C direction. The conservation of aromatic amino acids at the C-terminal positions, namely Tyr at 159 and Phe at 161, is evident. (B) Rate of hydrolysis of the *p*-nitrophenyl palmitate substrate analog by PagP mutants was monitored by the release of the chromogenic substrate *p*-nitrophenol at 405 nm. The enzyme activity was determined by fitting the linear segment of the rate plot, as reported previously (2). The mutants are named based on the residues present at the 160th and 161st positions. PagP-ΔF^{161} mutant is labeled as ΔF. Note that by and large, the activity of all the mutants is comparable to the WT

protein. (C) Gel mobility shift and protection against proteolysis by proteinase K (PK), as evidence of the folded state (in DPC micelles) of representative PagP variants from the PagP-X^{161} mutant library (top panel) and the PagP-X^{160} mutant library (bottom panel). Folded (F) and unfolded (U) proteins differ in their gel mobilities and the well-folded protein shows resistance to PK digestion. The terminal residue deletion mutants PagP-ΔF and PagP-ΔQF lack a prominent gel mobility shift in the folded protein and show an increased susceptibility to proteolysis, indicating significant destabilization of the folded barrel. A dotted line has been used to separate independent sections of the PagP-ΔF gel (top right panel) that are presented together as a composite figure. Molecular weights of protein standards in kDa, are provided on the left. PK: proteinase K added (+) or absent (–). Mutants are labeled using the single letter code for each amino acid, followed by the residue number (in superscript) at which the substitution has been carried out. Also see Figures S5 and S7 for the complete gels of all PagP-X^{160} and PagP-X^{161} mutants.

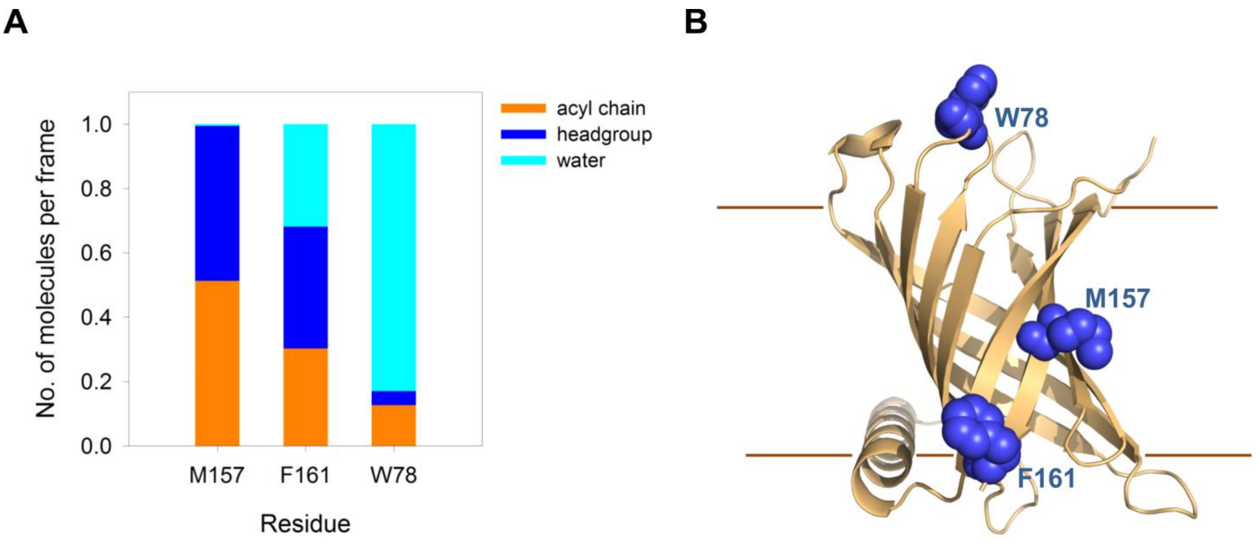

Figure S2. Vicinity analysis of PagP-WT using MD simulation. (A) Occupancy data of acyl chain, lipid headgroup and water molecules, at a vicinity of 5Å, presented for three different residues of PagP. The midplane residue of the terminal strand, M^{157}, shows characteristics typical of a buried residue. The terminal interface residue, F^{161} shows moderate occupancy for all three molecule types. The solvent-exposed residue W^{78}, located in the extracellular loop 2, serves as a negative control for lipid occupancy with maximal presence of water molecules in its 5Å vicinity. (B) Crystal structure of PagP from *E. coli* (PDB ID: 3GP6 (3)), rendered as a ribbon diagram, highlighting the three residues (blue spheres) for which the vicinity analysis is depicted in panel (A).

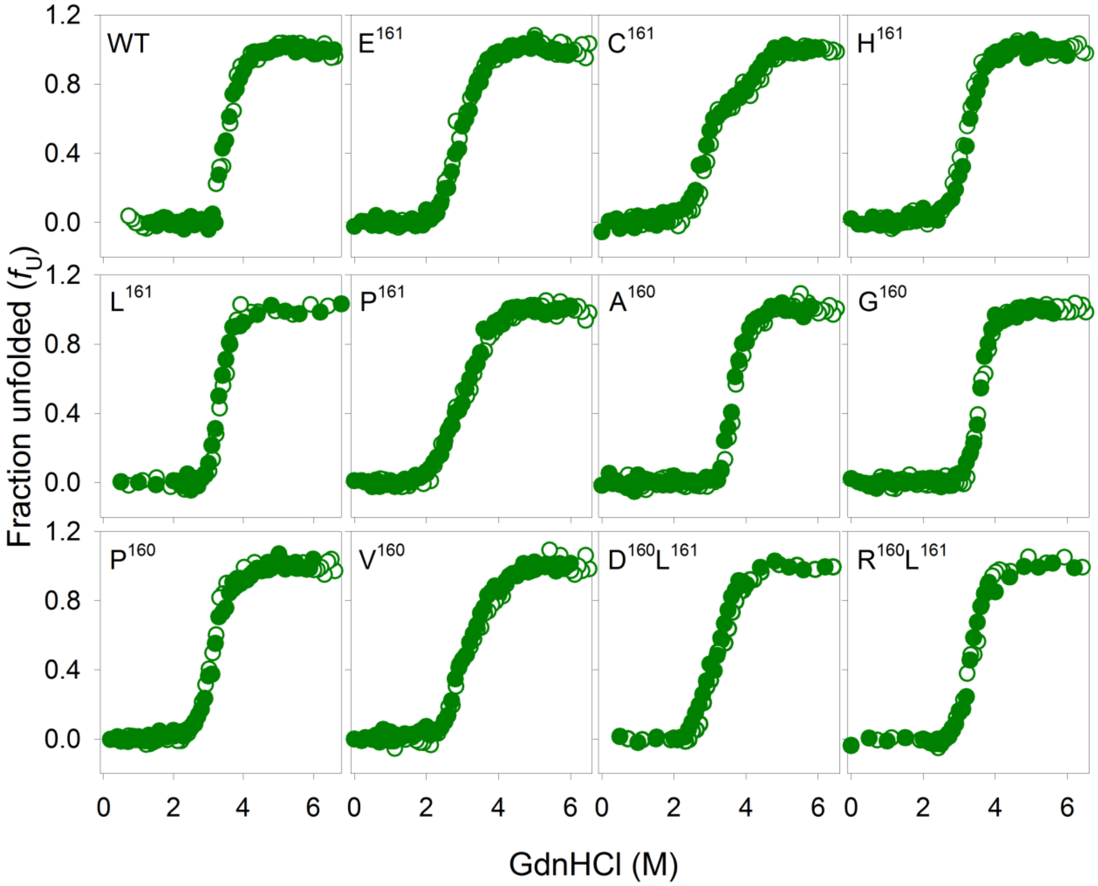

Figure S3. Representative equilibrium folding and unfolding profiles of PagP-X^{160} and PagP-X^{161} mutants to demonstrate path independence. Representative folding (open symbols) and unfolding (filled symbols) profiles monitored using change in fluorescence emission intensity of tryptophan at the λ_{em} of 344 nm, corresponding to the $\lambda_{em\text{-}max}$ of the folded protein. Data were corrected for buffer contributions, normalized between 0 and 1, and are presented as unfolded fractions (f_U) at each concentration of GdnHCl. Mutants are labeled using the single letter code for each amino acid, followed by the residue number (in superscript) at which the substitution has been carried out.

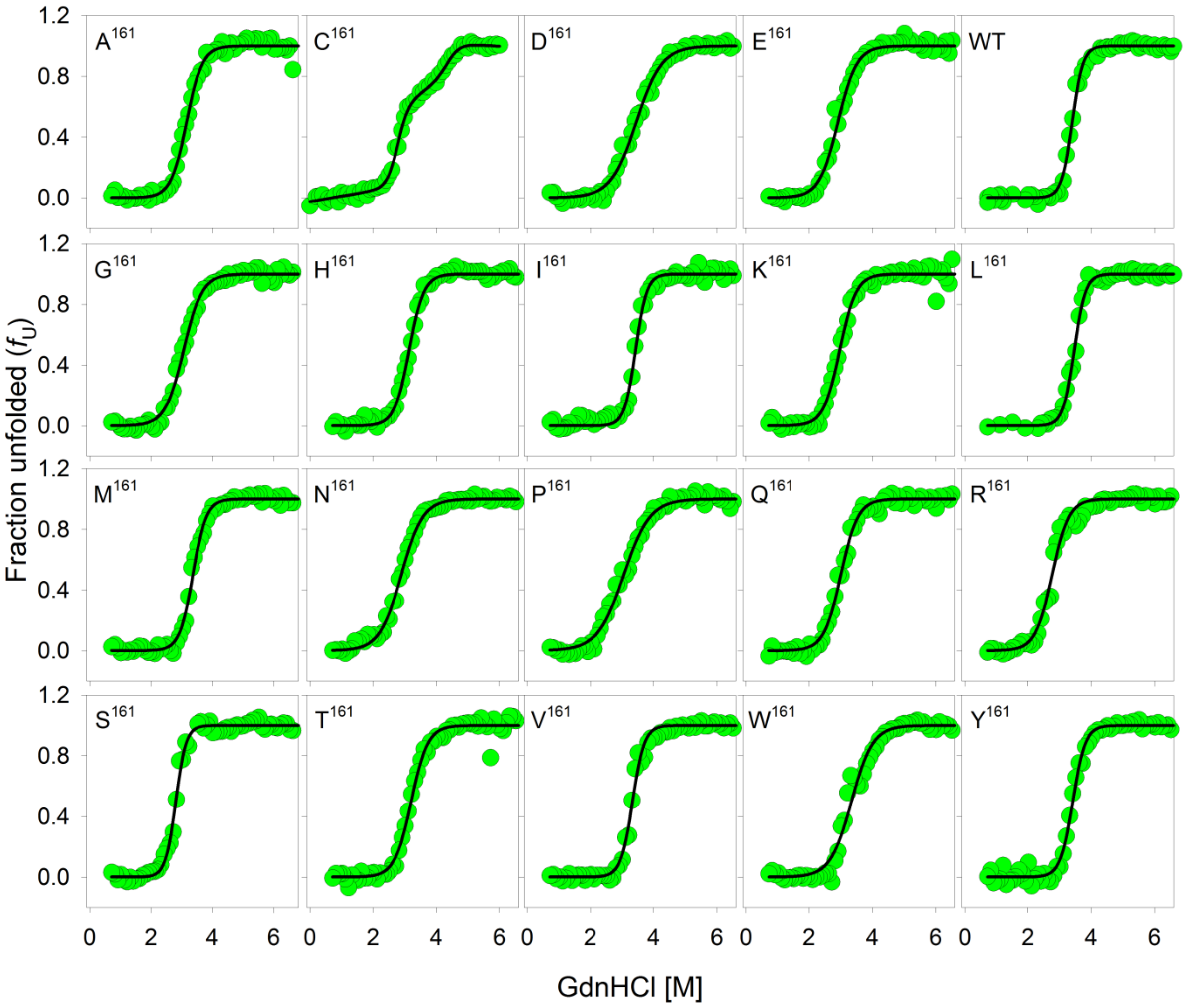

Figure S4. Equilibrium folding profiles of all PagP-X^{161} mutants. Unfolded protein fractions calculated by monitoring the change in fluorescence emission intensity of tryptophan at the $\lambda_{em\text{-}max}$ of 344 nm are plotted against denaturant concentration for all PagP-X^{161} mutants (X is any amino acid). Data for PagP-WT (F^{161}) is marked as WT. Data points are fitted to a two-state equation (4) (three-state equation (5) for PagP-C^{161}) to derive the thermodynamic parameters ΔG^0 and *m* value. Except for PagP-C^{161}, none of the other mutants could be fitted reliably to a three-state equation. Folding profiles are represented as green symbols and fits are shown as black solid lines. Mutants are labeled using the single letter code of the amino acid followed by the residue number (in superscript) at which the substitution has been carried out.

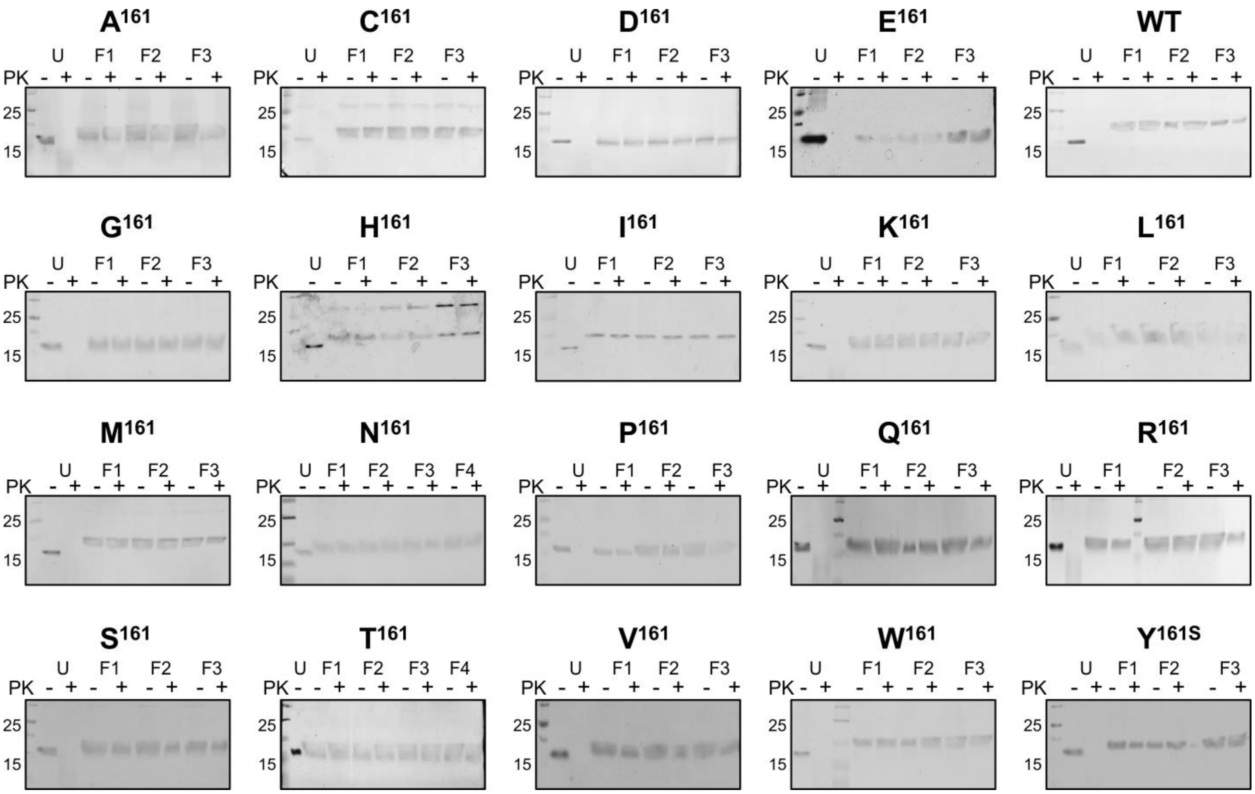

Figure S5. Folding of all PagP-X^{161} mutants monitored by gel shift assay. Gel mobility shift and protection against proteolysis by proteinase K (PK), as evidence of the folded state (in DPC micelles) of PagP variants from the PagP-X^{161} mutant library. Samples were checked on cold SDS-PAGE after the equilibrium fluorescence measurements, and these samples therefore contain trace amounts of GdnHCl used in the equilibrium experiments (note that the presence of GdnHCl in the sample interferes with the quality of SDS-PAGE profile). Folded (F1-F3 or F4) and unfolded (U) proteins differ in their gel mobility in most mutants; the folded protein additionally resists PK digestion. F1-F4 correspond to the first four samples in the equilibrium folding studies, and possess trace amounts of GdnHCl in the sample (0.7 M – 1.1 M). Note that while differences in electrophoretic mobility may not always indicate that PagP is folded (for example, see D^{161}), resistance to proteolysis by PK is a reliable indicator of PagP folding.

Molecular weights of protein standards in kDa, are provided on the left. PK: proteinase K added (+) or absent (–). Mutants are labeled using the single letter code for each residue, followed by the residue number (in superscript) at which the substitution has been carried out. All images present the region spanning ~10 kDa to ~37 kDa. The SDS-PAGE analysis of PagP- C^{161} (top row, second panel from the left) was carried out in a reducing environment. Hence, the small population of dimers seen in the gel (at ~30 kDa) may result from spontaneous PagP dimerization due to probable tertiary interactions, and is less likely to be the result of a disulfide bond. Note that several variants of PagP including PagP- H^{161} mutant (second row from the top, second panel from the left) exhibit dimer formation.

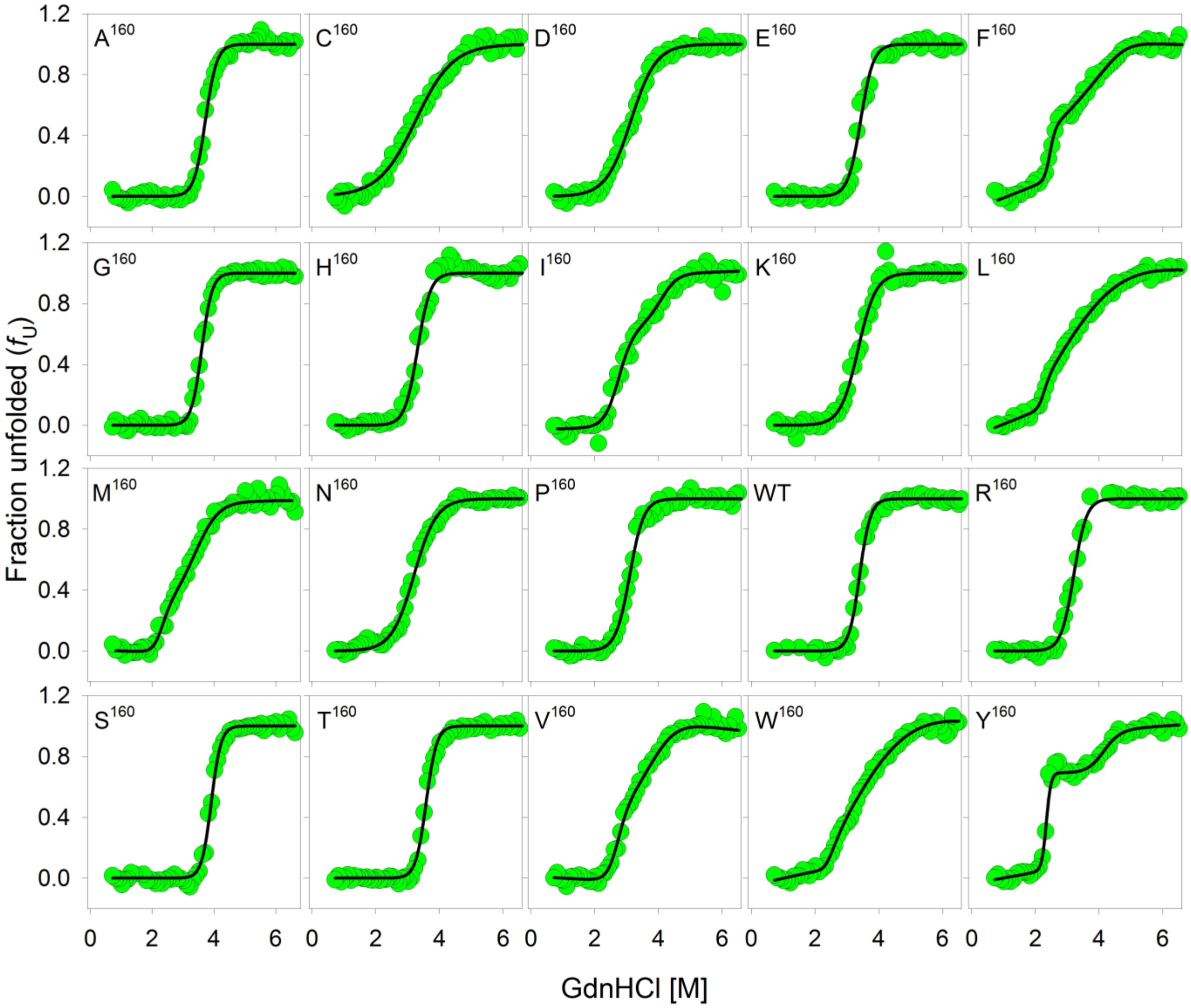

Figure S6. Equilibrium folding profiles of all PagP-X^{160}F^{161} mutants. Unfolded protein fractions calculated by monitoring the change in fluorescence emission intensity of tryptophan at the $\lambda_{em\text{-}max}$ of 344 nm are plotted against denaturant concentration for all PagP-X^{160}F^{161} mutants (X is any amino acid). Data for PagP-WT (Q^{160}) is marked as WT. Data points are fitted to a two-state equation (4) (three-state equation (5) for residues F, I, L, M, V, W, Y) to derive the thermodynamic parameters ΔG^0 and m value. Folding profiles are represented as green symbols and fits are shown as black solid lines. Mutants are labeled using the single letter code of the amino acid followed by the residue number (in superscript) at which the substitution has been carried out.

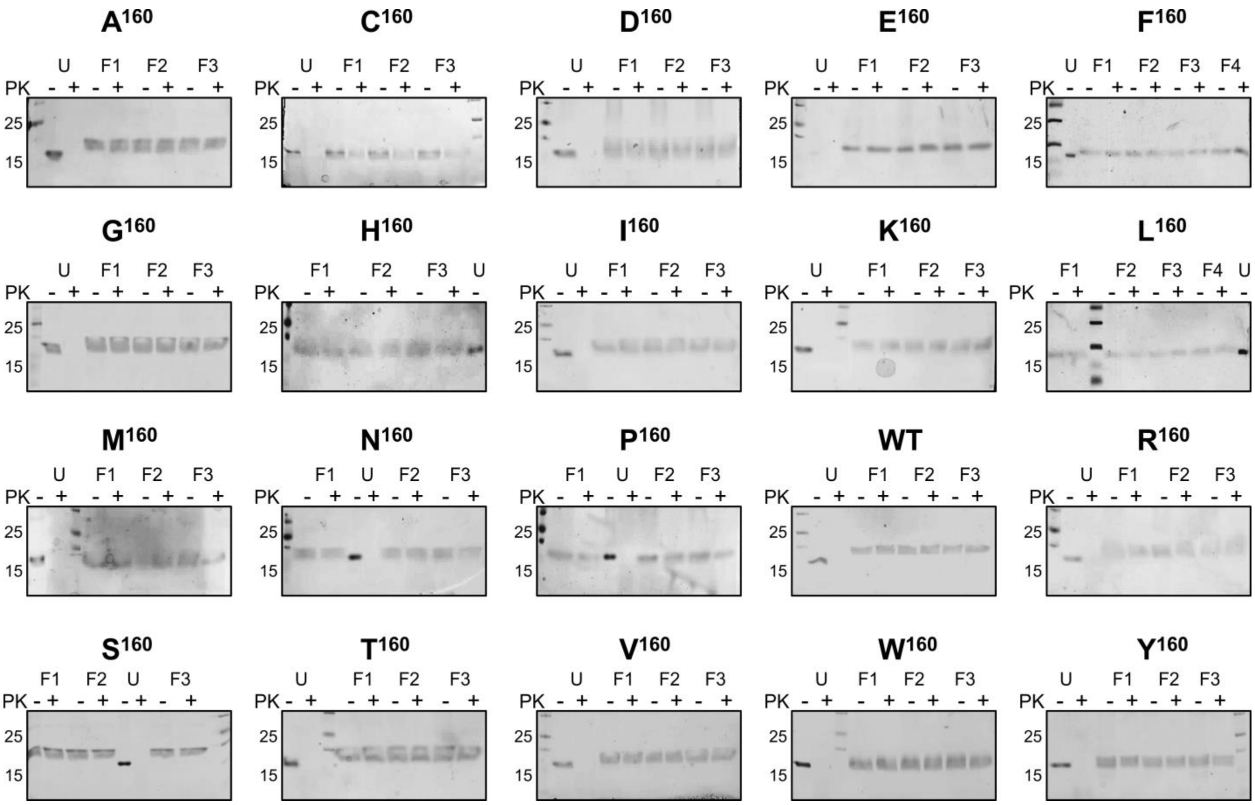

Figure S7. Folding of all PagP-X^{160} mutants monitored by gel shift assay. Gel mobility shift and protection against proteolysis by proteinase K (PK), as evidence of the folded state (in DPC micelles) of PagP variants from the PagP-X^{160} mutant library. Note that the samples also contain trace amounts of GdnHCl (refer to Figure S5 legend). Folded (F1-F3 or F4) and unfolded (U) proteins differ in their gel mobility in most mutants; the folded protein additionally resists PK digestion. F1-F4 correspond to the first four samples in the equilibrium folding studies, and possess trace amounts of GdnHCl in the sample (0.7 M – 1.1 M). Note that while differences in electrophoretic mobility may not always indicate that PagP is folded (for example, see L^{160}), resistance to proteolysis by PK is a reliable indicator of PagP folding. Molecular weights of protein standards in kDa, are provided on the left. PK: proteinase K added (+) or absent (–). Mutants are labeled using the single letter code for each residue, followed by the residue number (in superscript). All images present the region spanning ~10 kDa to ~37 kDa.

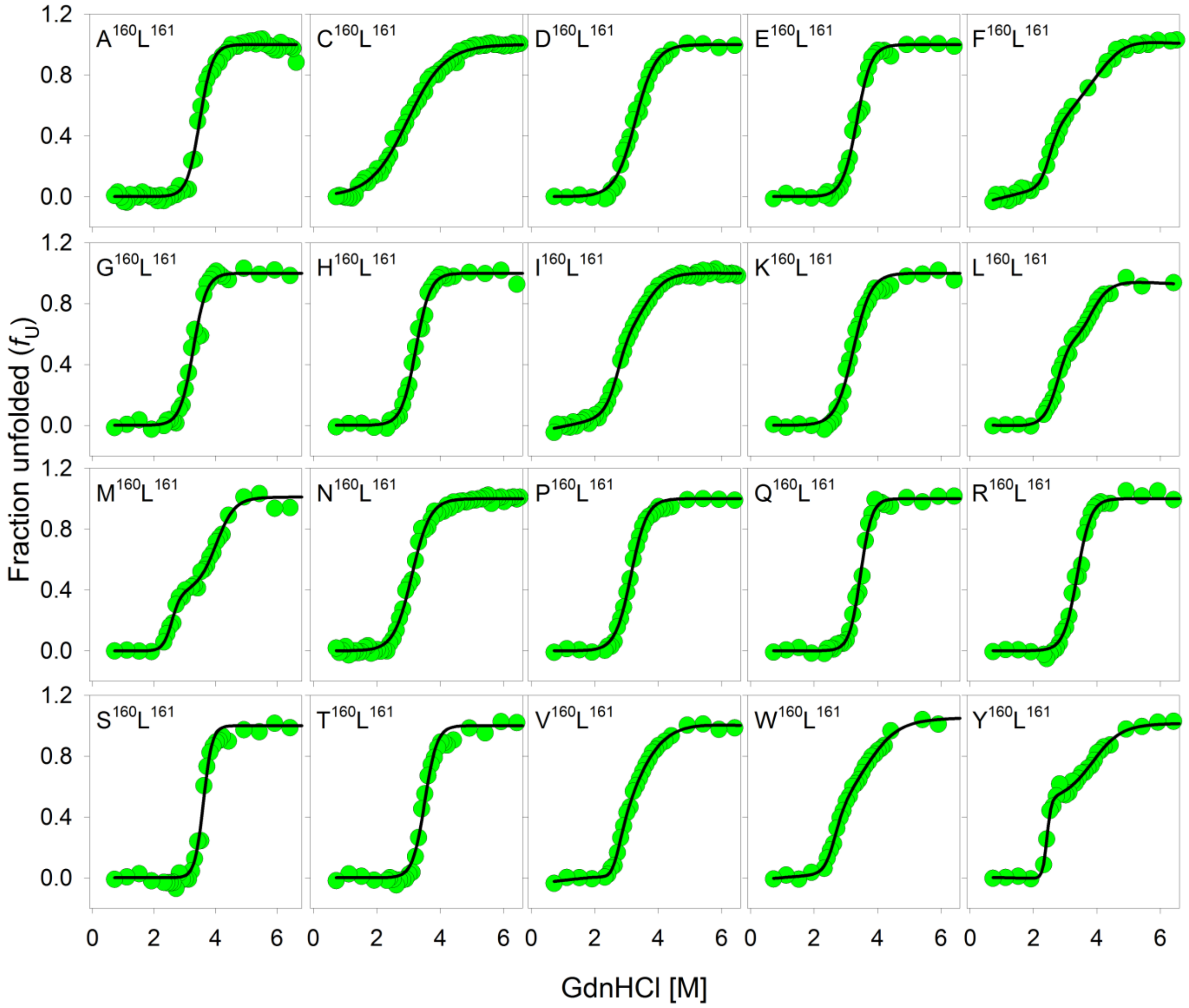

Figure S8. Equilibrium folding profiles of PagP-X^{160}L^{161} mutants. Unfolded protein fractions calculated by monitoring the change in fluorescence emission intensity of tryptophan at the $\lambda_{em\text{-}max}$ of 344 nm are plotted against denaturant concentration for all PagP-X^{160}L^{161} mutants (X is any amino acid). Data points are fitted to a two-state equation (4) (three-state equation (5) for penultimate residues F, I, L, M, V, W, Y) to derive the thermodynamic parameters ΔG^0 and *m* value. Folding profiles are represented as green symbols and fits are shown as black solid lines. Mutants are labeled using the single letter code of the amino acid followed by the residue number (in superscript) at which the substitution has been carried out.

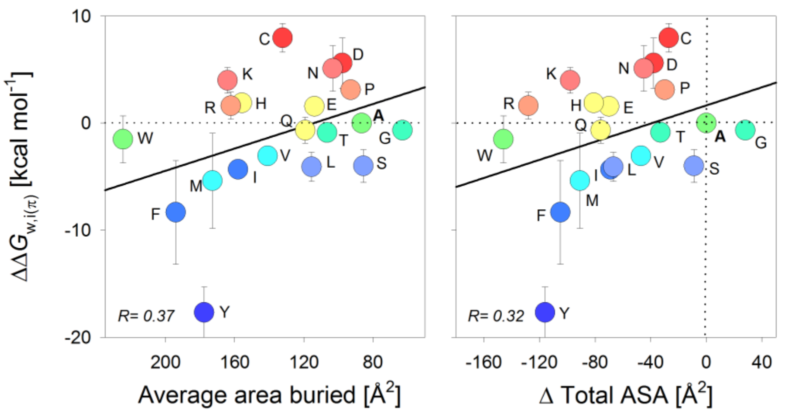

Figure S9. (Continued from Figure 3 of the main text) Correlation plots of free energy of penultimate protein-facing interface residue with change in ASA. Correlation between $\Delta\Delta G^0_{w,i(\pi)}$ and empirical parameters describing the change in ASA are shown as scatter plots. $\Delta\Delta G^0_{w,i(\pi)}$ values are averaged from PagP-$X^{160}F^{161}$ and PagP-$X^{160}L^{161}$ mutant libraries (error bars represent deviation from this average). Linear fits to the correlation are represented as solid black lines. Points that are excluded from the fits are shown as square symbols. The color code for the scatter plots is retained from Figure 3A of the main text.

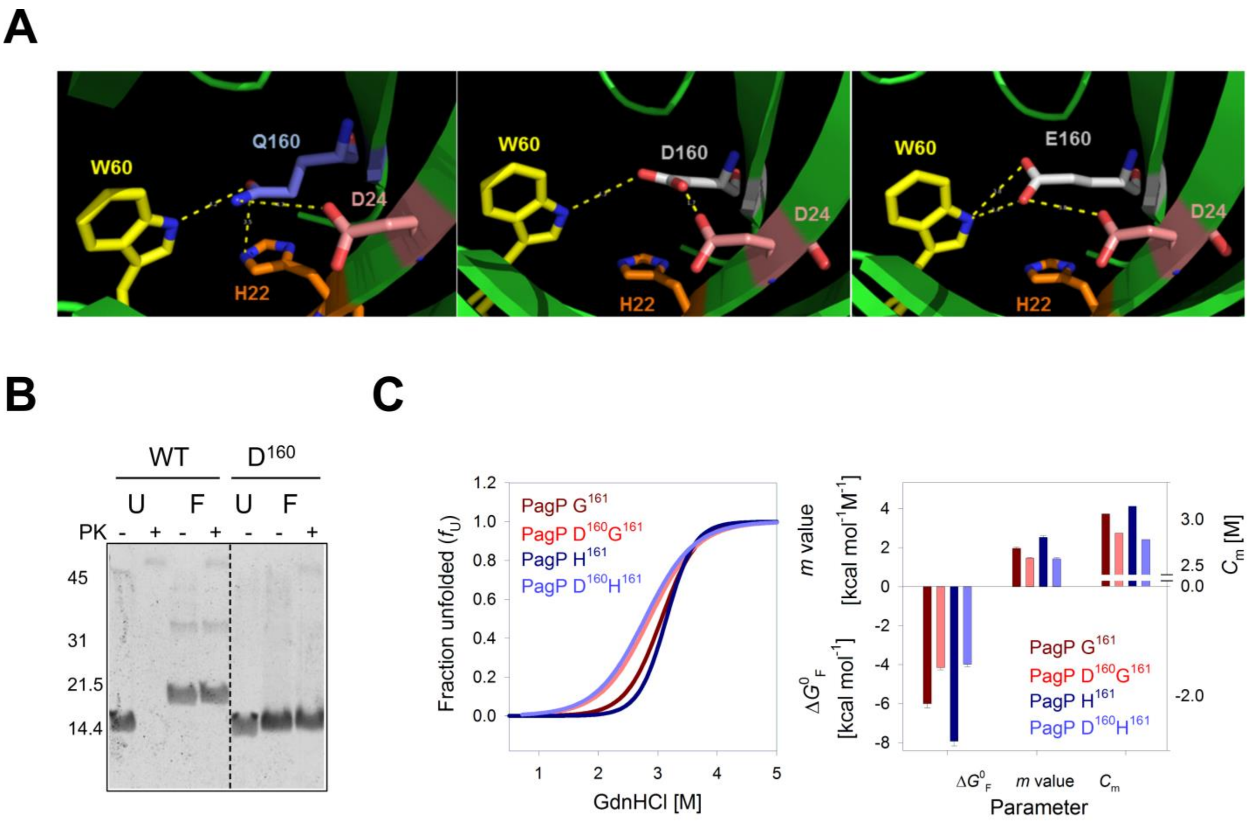

Figure S10. Effect of the Q→D^{160} mutation on the folding and stability of PagP. (A) The crystal structure of *E. coli* PagP (PDB ID: 1THQ) was rendered in PyMol (6) to generate the wire and stick model of Q160 (left, blue sticks) and residues H^{22} (orange), D^{24} (pink) and W^{60} (yellow) present in the vicinity of Q^{160}. The residue at position 160 was then mutated *in silico* to D (middle panel, grey sticks) and E (right panel, grey sticks) to monitor changes in the local interaction(s). Placing the short negatively charged side chain of Asp (middle panel) may cause electrostatic repulsion with the proximal D^{24}. This effect is not apparent in the case of Glu (right panel) since its side chain is longer than Asp. A likely consequence of the additional C$^{\gamma}$H$_2$ in Glu is to minimize electrostatic repulsion between the two negatively charged side chains of D^{24} and E^{160}. All residues are annotated with their single letter code followed by the amino acid number based on the primary sequence of mature *E. coli* PagP. (B) SDS-PAGE gel of unboiled samples

of PagP-WT (lanes on the left) showing gel mobility shift upon folding and resistance to proteolysis. For PagP-D^{160} (lanes on the right), the retarded gel mobility is absent, but the protease resistance is intact. Further, the far-UV CD spectra (presented in Figure 6 of the main text), are comparable for PagP-D^{160}, PagP-E^{160}, and PagP-WT. It is likely that barrel packing might be locally altered in the vicinity of D^{160} for this mutant, due to which the gel mobility is affected. Molecular weight marker positions (in kDa) are indicated on the left; U: PagP unfolded using 8.0 M urea; R: PagP folded in 50 mM DPC. PK: proteinase K added (+) or absent (–). PK migrates above 45 kDa and is seen as a faint band in the gel. A minor population of PagP-WT exists as a folded, PK-resistant dimer in solution. Dotted lines separate different gel images that have been presented together for comparison. (C) Unfolded protein fractions calculated using fluorescence emission intensity at the $\lambda_{em\text{-}max}$ of 344 nm are plotted against denaturant concentration for all PagP-G^{161} (brown), PagP-D^{160}G^{161}(pink), PagP-H^{161} (blue) and PagP-D^{160}H^{161} (light blue). Data points are fitted to a two-state equation (4) (fits are shown in the left panel) to derive the thermodynamic parameters ΔG^0, m value and C_m (right panel). Both mutants containing D^{160} are significantly destabilized compared to their respective parent proteins that possess Gln at position 160. This destabilization is reflected in the lowered m value and the C_m, which gives rise to lowering of the ΔG^0 to ~4 kcal mol^{-1}.

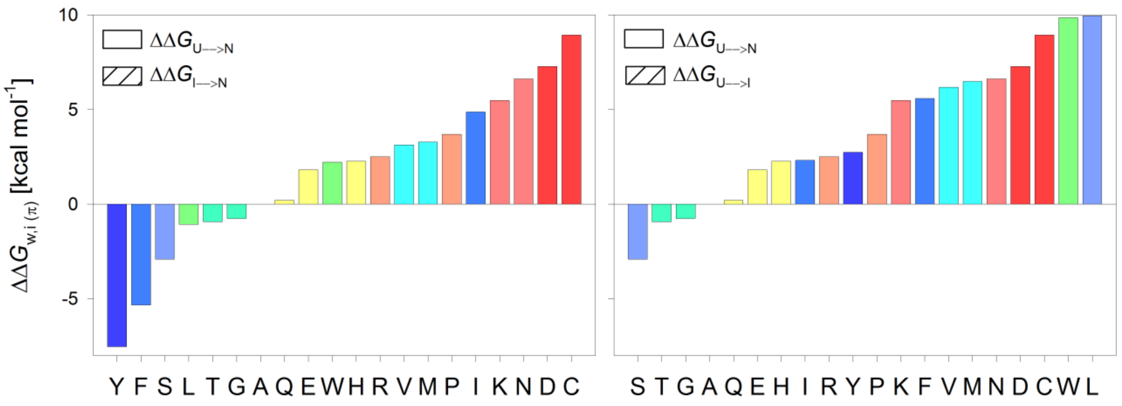

Figure S11. (Continued from Figure 3 of the main text) Partitioning free energy of penultimate protein-facing interface residue of PagP. Histograms generated by segregating the total change in folding free energy $\Delta\Delta G^0_{U\rightarrow N}$ for the PagP-X^{160}F^{161} mutants exhibiting three-state folding profiles into $\Delta\Delta G^0_{I\rightarrow N}$ (left panel) and $\Delta\Delta G^0_{U\rightarrow I}$ (right panel), presented along with $\Delta\Delta G^0_{U\rightarrow N}$ for the mutants with two-state folding profiles. Values from the three-state profiles are shown as a patterned fill in the histogram. The color code is retained from Figure 3A of the main text.

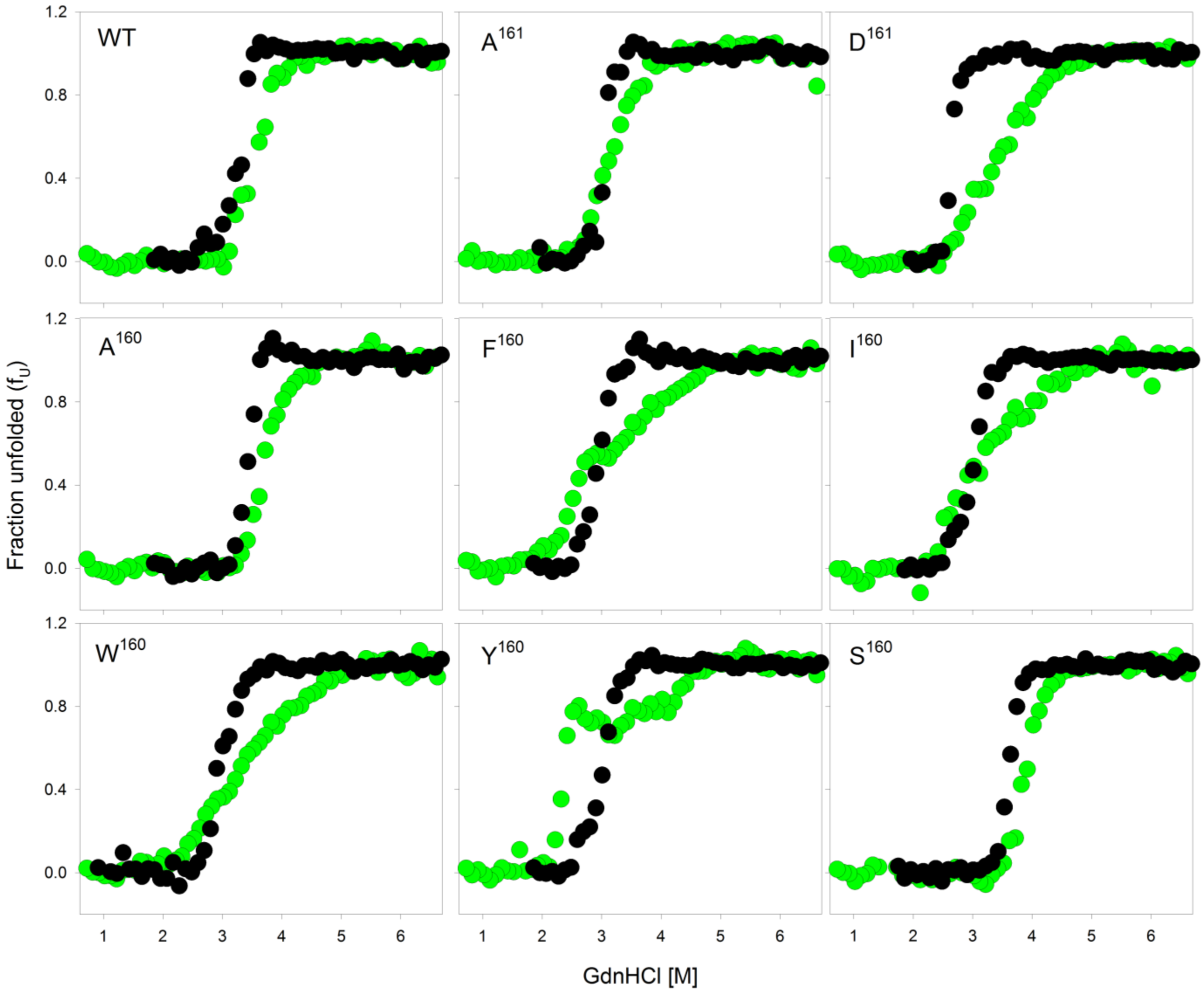

Figure S12. Comparison of equilibrium folding profiles in DPC micelles and DLPC SUVs. Representative folding profiles in DPC micelles (green symbols) and DLPC vesicles (black symbols) monitored using the change in fluorescence emission intensity at a λ_{em} of 344 nm, corresponding to the $\lambda_{em\text{-}max}$ of the folded protein. Shown here are data for select mutants from the PagP-X^{160} and PagP-X^{161} mutant series. It is evident that all mutants show a highly cooperative two-state transition in DLPC vesicles. Mutants showing three-state folding profiles in DPC micelles (including PagP-Y^{160}, lower middle panel) also exhibit two-state folding in

vesicles. Mutants are labeled using the single letter code of the amino acid followed by the residue number (in superscript) at which the substitution has been carried out.

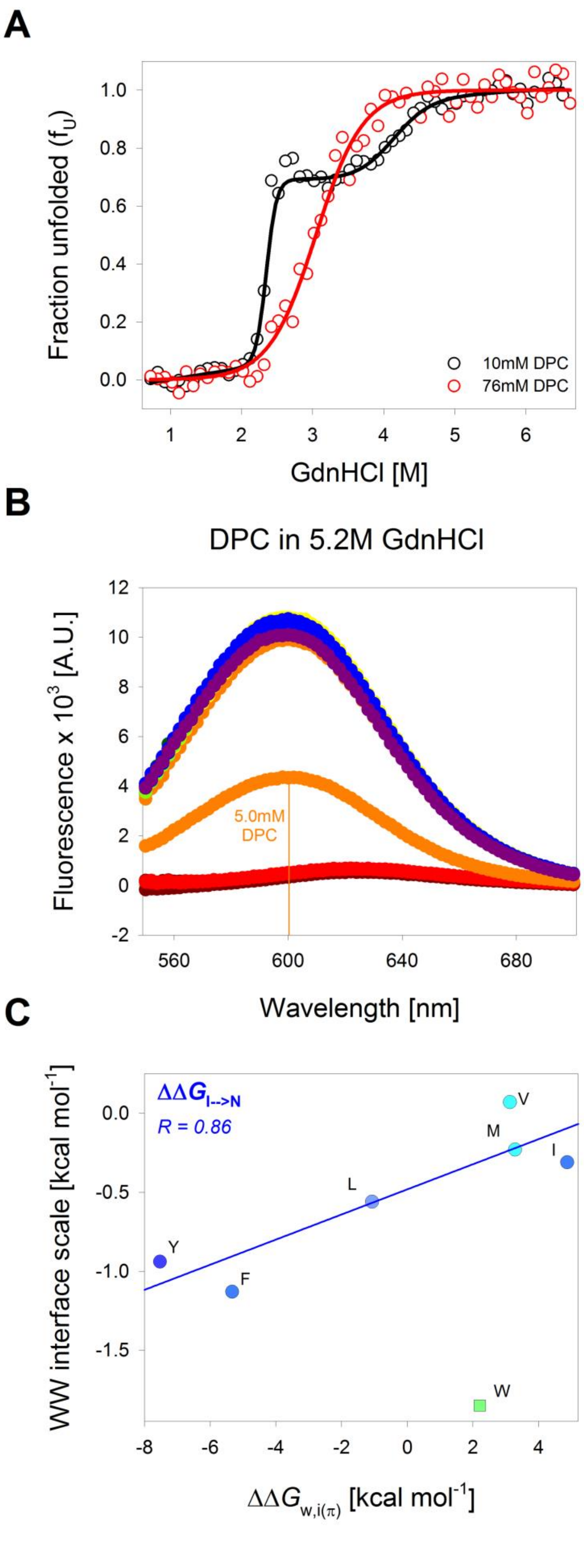

A
Fraction unfolded (f_U)
1.0
0.8
0.6
0.4
0.2
0.0
10mM DPC
76mM DPC
1
2
3
4
5
6
GdnHCl [M]
B
DPC in 5.2M GdnHCl
Fluorescence x 10^3 [A.U.]
12
10
8
6
4
2
0
-2
5.0mM
DPC
560
600
640
680
Wavelength [nm]
C
WW interface scale [kcal mol^-1]
ΔΔG_I-->N
R = 0.86
V
M
I
L
Y
F
W
0.0
-0.5
-1.0
-1.5
-8
-6
-4
-2
0
2
4
ΔΔG_w,i(π) [kcal mol^-1]

Figure S13. Equilibrium folding analysis of PagP-X^{160} hydrophobic residue substituents. (A) Representative profiles showing unfolded fractions for PagP-Y^{160}, calculated from fluorescence emission intensity values measured at the λ_{em-max} of 344 nm. f_U values are plotted for the respective denaturant concentrations at low (10 mM DPC, DPR ~ 3300:1, open black circles) and high (76 mM DPC, DPR ~ 25000:1, open black circles) DPC concentrations. Fits of the data to a three-state (black line) and two-state equation (red line) are shown. DPC and DLPC differ in the lipid packing efficiency due to the diacyl chain of DLPC. Hence, in high DPR of DPC, we find that the folding profile of PagP-Y^{160} (the mutant with the most prominent folding intermediate) is now two-state and we no longer observe an intermediate. When we examine the individual transitions, the ΔG^0 for the high DPR of DPC is closer to the $\Delta G^0_{U\rightarrow I}$ observed in low DPR (-9.3 kcal mol^{-1}), suggesting that the U→I transition may describe the global folding process. (B) Integrity of DPC micelles monitored by SYPRO® orange fluorescence. SYPRO® orange binds to hydrophobic regions with high affinity, which is accompanied by an increase in fluorescence intensity of the dye. This fluorescence profile shows a λ_{em-max} of 595 nm. Monomeric detergent that is solubilized in denaturant molecules does not bind SYPRO® orange efficiently, and the observed fluorescence emission profile is therefore red-shifted (λ_{em-max} = 622 nm) and of low intensity (red and maroon profiles). We observed an increase in the fluorescence intensity of SYPRO® orange with change in DPC concentration from 0.5 mM to 20 mM, prepared in a solution of 20 mM Tris-HCl pH 9.5 containing 5.2 M GdnHCl. The CMC of DPC in water or Tris-HCl is ~2 mM. The CMC is increased to ~5 mM in the presence of 5.2 M GdnHCl. All the fluorescence spectra recorded above a micellar concentration of 5 mM DPC (orange symbols, highlighted by drop line), show a blue shift and an increase in emission intensity. This suggests that at concentrations beyond 10 mM DPC (cluster of emission spectra from dark orange for 7.5 mM DPC to purple for 20 mM DPC), the micelles are intact even at a

high denaturant concentration of 5.2 M GdnHCl. All our experiments are carried out in 10 mM DPC, PagP is fully unfolded at GdnHCl concentrations of ~4.5 M. We thus confirm the integrity of the DPC micelles throughout the denaturant range used in our equilibrium folding measurements. Our measurements also confirm that PagP unfolds well before the micelle structures are solubilized by GdnHCl. (C) Correlation between $\Delta\Delta G_{w,i(\pi)}$ obtained for the I→N transition for the PagP-X^{160} mutants and Wimley-White interface sale. We plotted the $\Delta\Delta G_{w,i(\pi)}$ obtained for the I→N transition for the seven PagP-X^{160} mutants showing three-state folding, with the partition free energy obtained from the Wimley-White interface sale. A linear fit with a high regression coefficient (solid blue line, R = 0.86) was obtained for the correlation. Points that are excluded from the fits are shown as square symbols. The color code for the scatter plot is retained from Figure 3A of the main text. The correlation indicates that the I→N transition for the PagP-X^{160} three-state mutants is a function of the interface hydrophobicity of the residue at position 160.

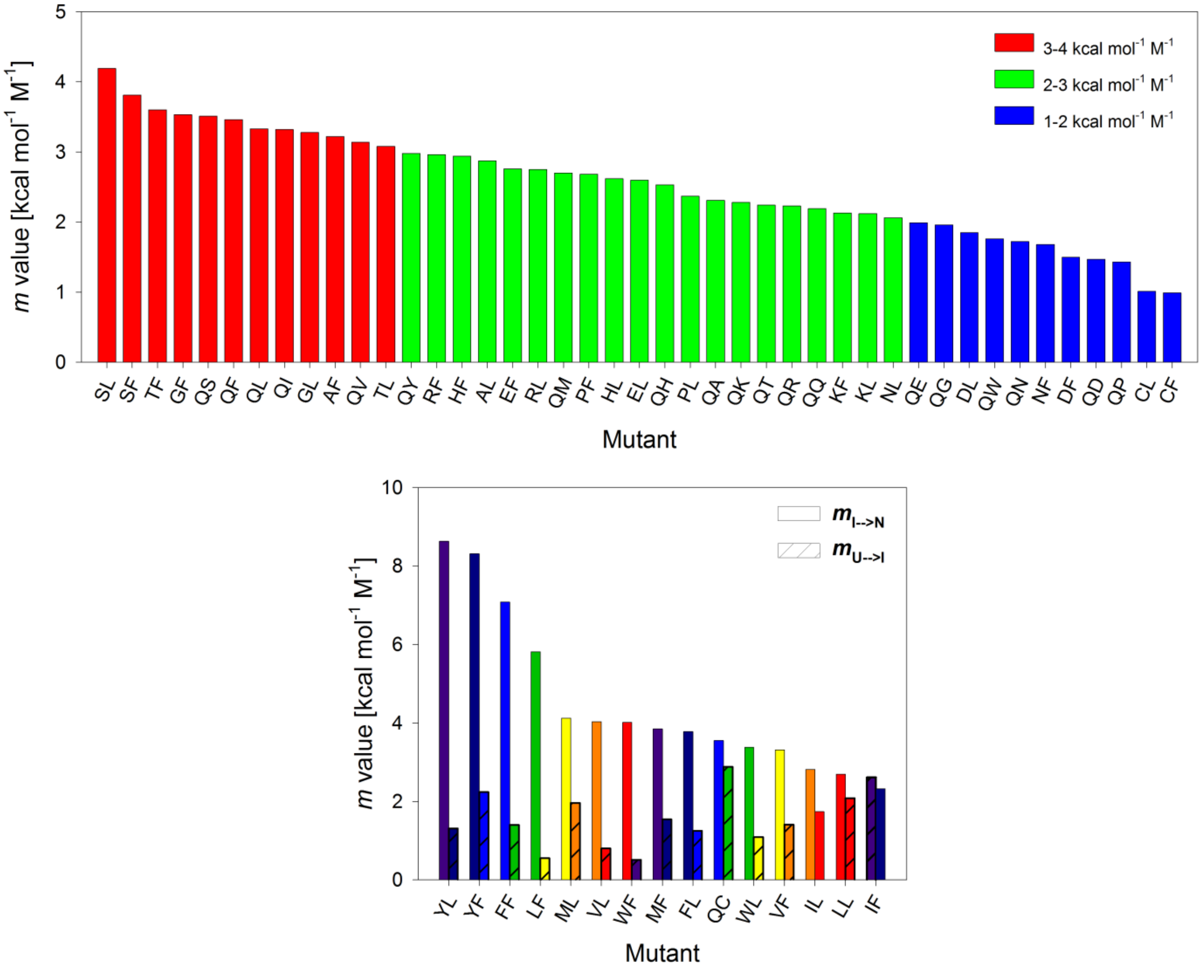

Figure S14. Summary of *m* values obtained for each mutant characterized in this study. Histograms depicting the *m* value derived from equilibrium folding measurements of PagP-X^{160} and PagP-X^{161} variants. The color code is based on the *m* value of the mutants. The *m* value for the mutants that show two-state folding profiles (upper panel) are divided into high (3-4 kcal mol^{-1} M^{-1}, red circles), moderate (2-3 kcal mol^{-1} M^{-1}, green circles) and low (1-2 kcal mol^{-1} M^{-1}, blue circles) *m* values. Data for the three-state mutants (lower panel) are colored using a rainbow scheme with the $m_{I \rightarrow N}$ (filled histogram) and the $m_{U \rightarrow I}$ (patterned histogram) plotted separately for each mutant. Mutants are annotated using the single letter codes of the amino acids

occupying positions 160 and 161 (e.g., PagP-C^{161} is represented as QC; PagP-WT is QF). A majority of the mutants with two-state profiles (upper panel) have moderate *m* value (2-3 kcal mol^{-1} M^{-1}), which is marginally lower than PagP-WT (*m* value of 3.5 kcal mol^{-1} M^{-1}). We observe a larger variation in *m* values of mutants showing three-state folding profiles, with PagP-YL displaying a $m_{I\rightarrow N}$ of 8.6 kcal mol^{-1} M^{-1} and PagP-WF showing a $m_{U\rightarrow I}$ of 0.5 kcal mol^{-1} M^{-1}. In all cases, we observed that $m_{I\rightarrow N}$ is larger than $m_{U\rightarrow I}$.

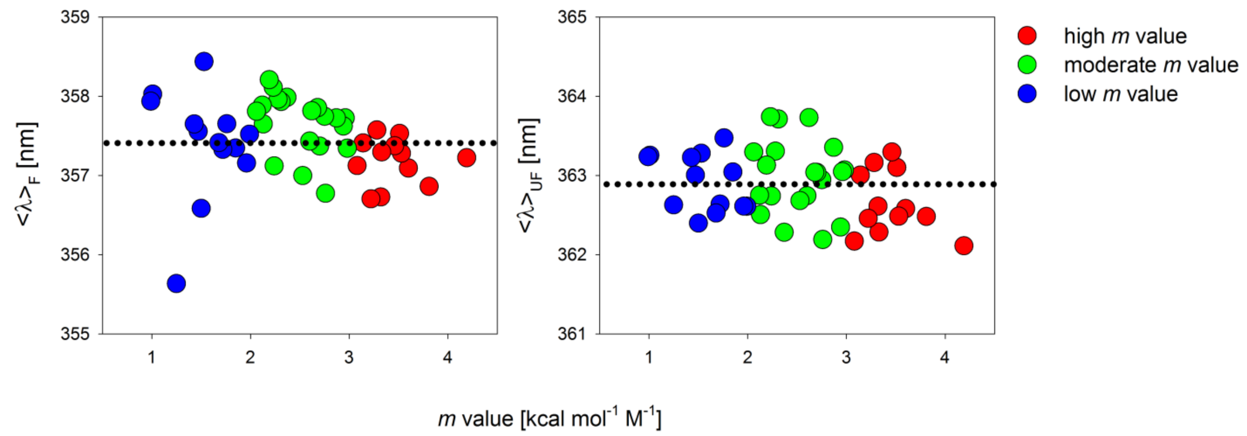

Figure S15. Correlation plot of the average wavelength values for PagP against the equilibrium folding *m* value. Both the correlation plots map the average wavelength of the folded states ($<\lambda>_F$, left panel) and average wavelength of the unfolded states ($<\lambda>_{UF}$, right panel) along the *y*-axis for each of the two state mutants from the PagP-X^{160} and PagP-X^{161} series. These <λ> values are compared with the corresponding *m* value measured for each mutant in DPC micelles. The $<\lambda>_F$ (average value ≈ 357.5 nm) as well as the $<\lambda>_{UF}$ (average value ≈ 363 nm) show a subtle blue shift when the *m* value increases. This effect is nullified when we calculate Δ<λ>, which remains constant across mutants (see Figure 6 of the main text). The scatter plot is colored based on the *m* value of the mutants, with the two state mutants divided into high (3-4 kcal mol^{-1} M^{-1}, red circles), moderate (2-3 kcal mol^{-1} M^{-1}, green circles) and low (1-2 kcal mol^{-1} M^{-1}, blue circles) *m* values.

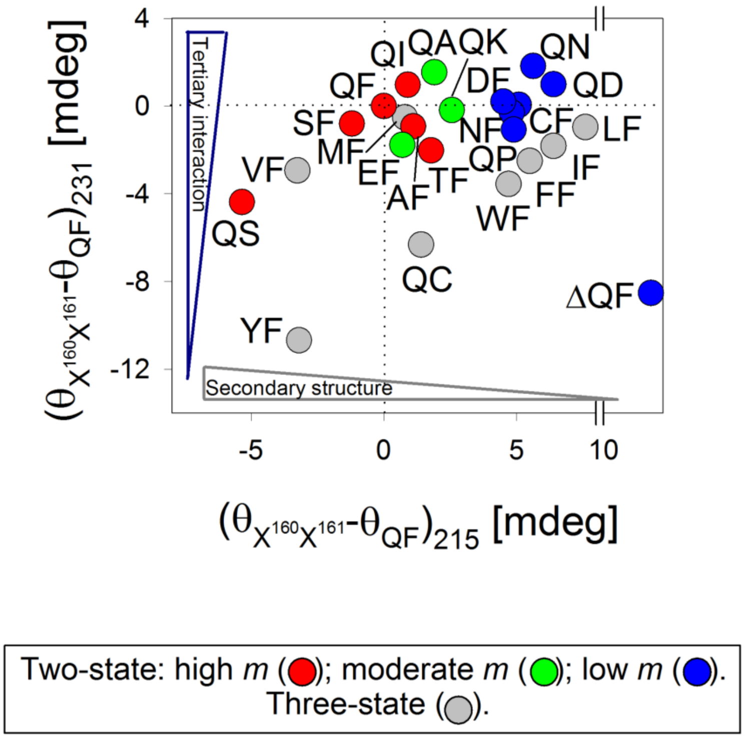

Figure S16. Correlation between secondary structure and observed tertiary CD for PagP upon folding in DPC micelles. Correlation plot between θ_{215} and θ_{231} for all the mutants described in Figure 6A, normalized with respect to PagP-WT (QF). The scatter plot is colored based on the *m* value of the mutants, with the two state mutants divided into high (3-4 kcal mol^{-1} M^{-1}, red circles), moderate (2-3 kcal mol^{-1} M^{-1}, green circles) and low (1-2 kcal mol^{-1} M^{-1}, blue circles) *m* values, while the three state mutants are represented as grey circles. Mutants are annotated using the single letter codes of the amino acids occupying positions 160 and 161 (eg., PagP-C^{161} is represented as QC). The θ_{231} value varies over a narrower range compared to θ_{215} across the mutants. We observe that for the two state mutants, the *m* values show reasonable clustering based on the secondary structure content (θ_{215}), while there is no apparent correlation to the tertiary CD (θ_{231}).

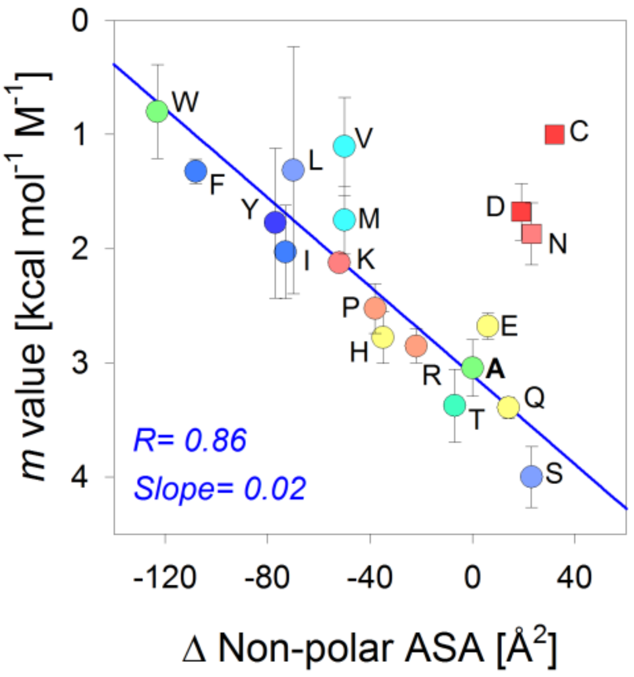

Figure S17. Correlation between $m_{U\rightarrow I}$ for the PagP X^{160} mutants and the change in non-polar ASA of the guest residue. We plotted the *m* value obtained for all the PagP-X^{160}F^{161} and PagP-X^{160}L^{161} mutants against the change in ΔASA (non-polar) with respect to Ala. Here, only the *m* value for the U→I transition for the mutants that showed a three state transition while folding, and the *m* value obtained for the U→N transition for two state transitions were considered. An inverse fit with a high regression coefficient (solid blue line, R = 0.86) is obtained for the correlation. Points that are excluded from the fits are shown as square symbols. The color code for the scatter plot is retained from Figure 5. The strong correlation indicates that global folding process for the PagP-X^{160} mutants is driven by the change in non-polar ASA of the amino acid side chain.

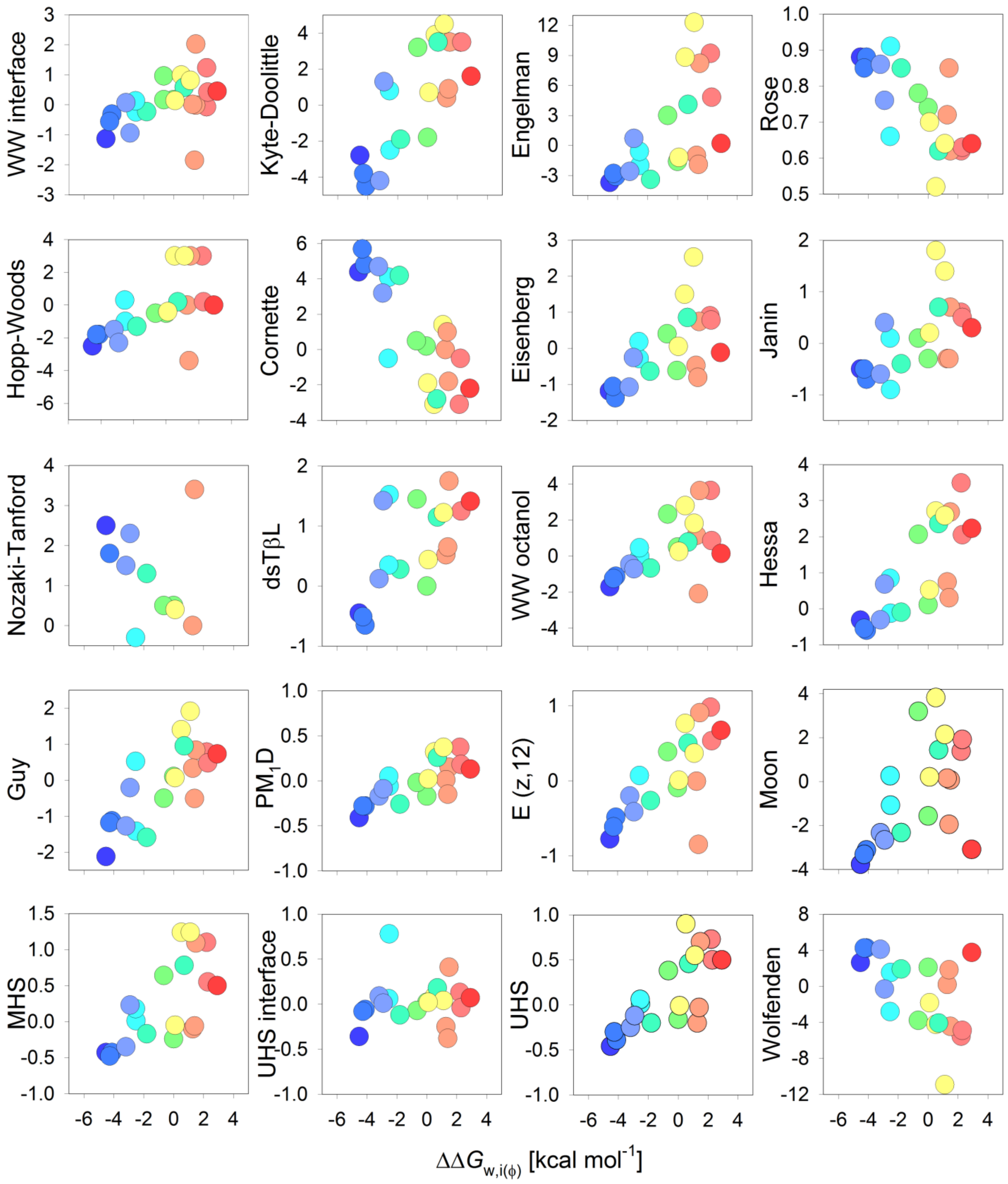

Figure S18. Comparison of $\Delta\Delta G_{w,i(\phi)}$ interface hydrophobicity scale with previously reported scales. Correlation plots generated by mapping the $\Delta\Delta G_{w,i(\phi)}$ interface hydrophobicity

scale with 20 previously reported scales including the scales discussed in Figure 7 of the main text. The color code for the scatter plot is retained from Figure 2A of the main text. In most of the correlations, the hydrophobic residues (shades of blue) are clustered at one end and the hydrophilic residues (shades of red) are clustered at the other, indicating strong correlation between our scale and reported values. WW interface: Wimley-White interface scale (7); Kyte-Doolittle: Kyte and Doolittle scale (8); Engelman: Goldman, Engelman and Steitz scale (9); Rose: Rose hydrophobicity scale (10); Hopp-Woods: Hopp and Woods scale (11); Cornette: Cornette hydrophobicity scale (12); Eisenberg: Eisenberg, Weiss and Terwilliger scale (13); Janin: Janin hydrophobicity scale (14); Nozaki-Tanford: Nozaki and Tanford scale (15); dsTβL: dsTβL scale by Elazar *et. al* (16); WW octanol: Wimley-White octanol scale (17); Hessa: Biological translocon scale (18); Guy: Guy hydrophobicity scale (19); PM_1D: Punta and Maritan 1D scale (20); E(z,12): E_z potential by Senes *et. al* (21); Moon: Moon-Fleming whole-protein scale (5); MHS: Mammalian hydrophobicity scale (22); UHS interface: Unified interface hydrophobicity scale (22); UHS : Unified hydrophobicity scale (22); Wolfenden: Wolfenden hydrophobicity scale (23).

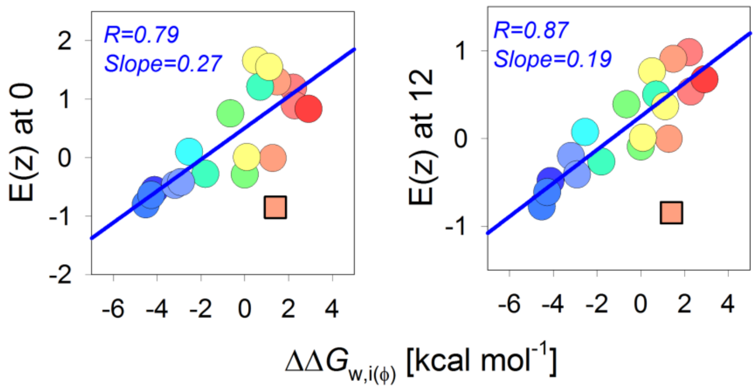

Figure S19. Correlation of $\Delta\Delta G_{w,i(\phi)}$ interface scale with the E(z) potential hydrophobicity scale. Correlation plots generated by mapping the $\Delta\Delta G_{w,i(\phi)}$ interface hydrophobicity scale with the E(z) potential hydrophobicity scale (21). The color code for the scatter plot is retained from Figure 2A of the main text. We observe that the correlation is stronger (R = 0.87) for a membrane depth of z = 12, which is an indicator of membrane interface position. This particular observation is suggestive of the fact that F^{161} may be positioned at the water-lipid interface, as opposed to a buried location.

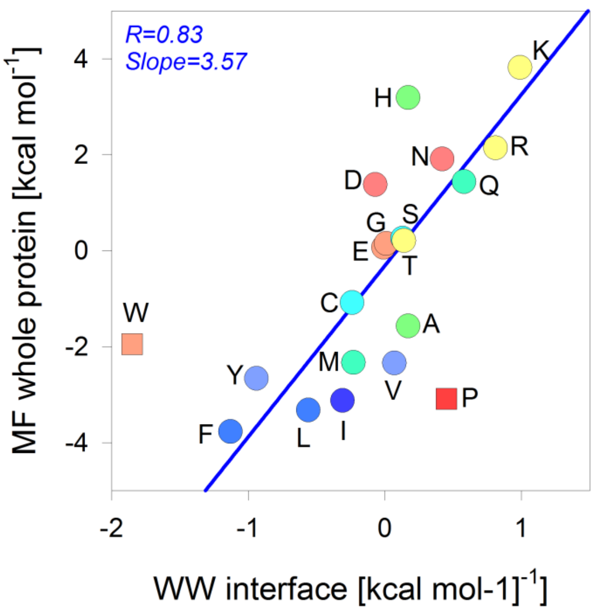

Figure S20. Correlation of Moon-Fleming whole protein hydrophobicity scale with the Wimley-White interface hydrophobicity scale. Correlation plot generated by mapping the Moon-Fleming whole protein hydrophobicity scale (5), measured for the membrane midplane with the Wimley-White interface hydrophobicity scale (7), reported for the water-lipid interface. The color code for the scatter plot is retained from Figure 2A of the main text. We observe that there is a strong correlation (R = 0.83) between the two scales, suggesting that similar side chain properties can govern partitioning into the hydrophobic core of the membrane as well as the water-lipid interface. Note that the magnitude of contribution of each side chain differs considerably between the interface position and the bilayer midplane, accounting for the slope of 3.57. A striking outlier in this comparison is the tryptophan residue, which possesses both hydrophobic and hydrophilic properties in its side chain.

Supplemental Tables

Table S1. List of primers used in this study.

**Mutant series**[#]	**Sequence (5'–3', reverse complement)**[@]
WT	GCTTTGTTAGCAGCCGGATCCTTAGAACTGGAAGCGCATCCAGGCAAAGTACAC
ΔF	GCTTTGTTAGCAGCCGGATCCTTATTACTGGAAGCGCATCCAGGCAAAGTACAC
ΔQF	GCTTTGTTAGCAGCCGGATCCTTAGAAGCGCATCCAGGCAAAGTACAC
Non-specific	GCTTTGTTAGCAGCCGGATCCTTA**NNNNNN**GAAGCGCATCCAGGCAAAGTACAC
X^{161}	GCAGCCGGATCCTTA**NNA**CTGGAAGCGCATCCAGG
X^{161}	GCAGCCGGATCCTTA**NNG**CTGGAAGCGCATCCAGG
X^{161}	GCAGCCGGATCCTTA**NNC**CTGGAAGCGCATCCAGG
X^{161}	GCAGCCGGATCCTTA**NNT**CTGGAAGCGCATCCAGG
X^{160}	GCCGGATCCTTAGAA**NNA**GAAGCGCATCCAGGCAAAG
X^{160}	GCCGGATCCTTAGAA**NNG**GAAGCGCATCCAGGCAAAG
X^{160}	GCCGGATCCTTAGAA**NNC**GAAGCGCATCCAGGCAAAG
X^{160}	GCCGGATCCTTAGAA**NNT**GAAGCGCATCCAGGCAAAG
$X^{160}L^{161}$	GCAGCCGGATCCTTACAG**ANN**GAAGCGCATCCAGGCAAAG
$X^{160}L^{161}$	GCAGCCGGATCCTTACAG**GNN**GAAGCGCATCCAGGCAAAG
$X^{160}L^{161}$	GCAGCCGGATCCTTACAG**CNN**GAAGCGCATCCAGGCAAAG
$X^{160}L^{161}$	GCAGCCGGATCCTTACAG**TNN**GAAGCGCATCCAGGCAAAG

[#] WT: PagP-WT; ΔF: PagP-ΔF^{161}; ΔQF: PagP-Δ$Q^{160}F^{161}$; X^{161}: PagP-$Q^{160}X^{161}$; X^{160}: PagP-$X^{160}F^{161}$; $X^{160}L^{161}$: PagP-$X^{160}L^{161}$.

[@]N: any nucleotide. The sequence of all mutants was confirmed using Sanger sequencing.

Table S2. Summary of Trp fluorescence lifetimes (<τ>) and anisotropy values (r) of representative mutants from the PagP-X^{160} and PagP-X^{161} mutant series.

Protein#	Lifetimes (ns)			Amplitudes			<τ> (ns)	χ^2	r
	τ1	τ2	τ3	α1	α2	α3			
**A^{160}-F**	0.77	3.40	6.78	0.26	0.57	0.18	**3.32**	1.07	**0.096**
**A^{160}-U**	0.59	2.36	5.86	0.37	0.51	0.12	**2.12**	1.13	**-**
**A^{160}-U(DPC)**	0.63	2.50	5.74	0.38	0.5	0.12	**2.17**	1.06	**0.049**
**E^{160}-F**	0.74	3.13	6.83	0.3	0.54	0.16	**3.00**	1.12	**0.096**
**E^{160}-U**	0.68	2.50	5.99	0.40	0.5	0.1	**2.10**	1.06	**-**
**E^{160}-U(DPC)**	0.80	2.41	4.54	0.35	0.5	0.15	**2.18**	1.12	**0.051**
**G^{160}-F**	0.82	3.49	6.77	0.25	0.52	0.22	**3.56**	1.08	**0.091**
**G^{160}-U**	0.64	2.49	6.02	0.39	0.5	0.11	**2.16**	1.09	**-**
**G^{160}-U(DPC)**	0.69	2.61	5.93	0.4	0.48	0.12	**2.23**	1.17	**0.048**
**H^{160}-F**	3.51	0.79	6.87	0.39	0.26	0.35	**3.96**	1.12	**-**
**H^{160}-U**	0.67	2.68	6.43	0.42	0.47	0.11	**2.23**	1.15	**-**
**H^{160}-U(DPC)**	0.67	2.60	5.96	0.41	0.46	0.13	**2.24**	1.18	**-**
**I^{160}-F**	0.73	3.32	6.58	0.25	0.55	0.2	**3.34**	1.13	**-**
**I^{160}-U**	0.70	2.57	6.08	0.41	0.49	0.1	**2.15**	1.18	**-**
**I^{160}-U(DPC)**	0.66	2.59	5.90	0.4	0.49	0.11	**2.17**	1.14	**-**
**K^{160}-F**	0.79	3.41	6.88	0.25	0.55	0.2	**3.46**	1.04	**0.095**
**K^{160}-U**	0.73	2.62	6.30	0.41	0.5	0.09	**2.17**	1.08	**-**
**K^{160}-U(DPC)**	0.70	2.63	5.93	0.41	0.49	0.11	**2.20**	1.19	**0.049**
**N^{160}-F**	0.76	3.28	6.45	0.24	0.51	0.25	**3.45**	1.07	**0.096**
**N^{160}-U**	0.77	2.68	6.23	0.42	0.48	0.1	**2.22**	1.16	**-**
**N^{160}-U(DPC)**	0.72	2.65	6.01	0.4	0.49	0.11	**2.22**	1.02	**0.049**

Protein	Lifetimes (ns)			Amplitudes			<τ> (ns)	χ^2	r
	τ1	τ2	τ3	α1	α2	α3			
**P^{160}-F**	0.85	3.64	6.88	0.29	0.49	0.23	**3.57**	0.98	**0.094**
**P^{160}-U**	0.60	2.55	6.21	0.41	0.48	0.11	**2.15**	1.15	**-**
**P^{160}-U(DPC)**	0.75	2.73	6.02	0.41	0.48	0.11	**2.29**	1.12	**0.047**
**R^{160}-F**	0.80	3.70	6.99	0.29	0.52	0.19	**3.50**	1.11	**-**
**R^{160}-U**	0.67	2.68	6.43	0.42	0.47	0.11	**2.23**	1.15	**-**
**R^{160}-U(DPC)**	0.60	2.55	6.05	0.41	0.45	0.14	**2.22**	1.11	**-**
**S^{160}-F**	0.79	3.31	6.73	0.27	0.57	0.17	**3.19**	1.16	**-**
**S^{160}-U**	0.69	2.47	5.92	0.4	0.49	0.1	**2.10**	1.03	**-**
**S^{160}-U(DPC)**	0.62	2.40	5.52	0.38	0.49	0.14	**2.15**	1.16	**-**
**W^{160}-F**	0.92	3.60	7.84	0.32	0.53	0.15	**3.36**	1.10	**-**
**W^{160}-U**	0.78	2.69	6.14	0.41	0.5	0.08	**2.19**	1.02	**-**
**W^{160}-U(DPC)**	0.65	2.49	5.59	0.37	0.5	0.13	**2.19**	1.14	**-**
**C^{161}-F**	0.72	3.04	6.28	0.26	0.52	0.22	**3.16**	1.12	**0.102**
**C^{161}-U**	0.67	2.25	4.19	0.31	0.48	0.2	**2.15**	1.02	**-**
**C^{161}-U(DPC)**	0.74	2.33	4.47	0.36	0.5	0.14	**2.04**	1.17	**0.068**
**D^{161}-F**	0.82	3.29	6.63	0.26	0.52	0.21	**3.36**	1.05	**0.097**
**D^{161}-U**	0.68	2.29	4.23	0.3	0.51	0.19	**2.17**	1.02	**-**
**D^{161}-U(DPC)**	0.72	2.41	4.63	0.35	0.54	0.11	**2.05**	1.09	**0.063**
**M^{161}-F**	0.77	3.21	6.69	0.25	0.55	0.2	**3.32**	1.07	**0.098**
**M^{161}-U**	0.66	2.25	4.18	0.3	0.5	0.2	**2.15**	1.04	**-**
**M^{161}-U(DPC)**	0.73	2.44	4.80	0.39	0.51	0.1	**2.01**	1.11	**0.063**
**P^{161}-F**	0.97	3.58	6.72	0.26	0.52	0.22	**3.57**	1.07	**0.098**
**P^{161}-U**	0.77	2.41	4.31	0.31	0.51	0.18	**2.25**	1.03	**-**

Protein	Lifetimes (ns)			Amplitudes			<τ> (ns)	χ^2	r
	τ1	τ2	τ3	α1	α2	α3			
**P^161-U(DPC)**	0.82	2.54	5.10	0.37	0.56	0.08	**2.11**	1.05	**0.063**
**S^161-F**	0.79	3.45	7.00	0.26	0.57	0.17	**3.37**	1.07	**0.097**
**S^161-U**	0.74	2.38	4.31	0.31	0.52	0.17	**2.2**	1.11	**-**
**S^161-U(DPC)**	0.73	2.46	4.72	0.33	0.56	0.11	**2.15**	1.11	**0.062**
**V^161-F**	0.69	3.26	6.74	0.25	0.57	0.18	**3.25**	1.04	**0.099**
**V^161-U**	0.77	2.40	4.39	0.3	0.53	0.17	**2.24**	1.17	**-**
**V^161-U(DPC)**	0.79	2.45	5.02	0.34	0.57	0.09	**2.10**	0.97	**0.061**
**W^161-F**	0.85	3.19	7.06	0.26	0.49	0.25	**3.55**	1.09	**0.109**
**W^161-U**	0.61	2.19	4.39	0.28	0.48	0.23	**2.26**	1.18	**-**
**W^161-U(DPC)**	0.78	2.55	5.24	0.38	0.51	0.1	**2.16**	1.12	**0.066**
**Y^161-F**	0.90	3.44	7.07	0.28	0.57	0.15	**3.29**	0.96	**0.098**
**Y^161-U**	0.67	2.29	4.30	0.32	0.51	0.18	**2.13**	1.08	**-**
**Y^161-U(DPC)**	0.65	2.28	4.62	0.35	0.53	0.12	**1.99**	1.07	**0.047**

[#]Mutants are labeled using the single letter code of the amino acid followed by the residue number (in superscript) at which the substitution has been carried out. F: PagP folded in 10 mM DPC; U: PagP unfolded using 8.0 M GdnHCl; U(DPC): PagP unfolded using 8.0 M GdnHCl containing 10 mM DPC.

Supplemental References

1. Crooks, G. E., Hon, G., Chandonia, J. M., and Brenner, S. E. (2004) WebLogo: a sequence logo generator *Genome Res.* **14**, 1188-1190
2. Iyer, B. R., and Mahalakshmi, R. (2015) Residue-Dependent Thermodynamic Cost and Barrel Plasticity Balances Activity in the PhoPQ-Activated Enzyme PagP of Salmonella typhimurium *Biochemistry* **54**, 5712-5722
3. Cuesta-Seijo, J. A., Neale, C., Khan, M. A., Moktar, J., Tran, C. D., Bishop, R. E., Pomes, R., and Prive, G. G. (2010) PagP crystallized from SDS/cosolvent reveals the route for phospholipid access to the hydrocarbon ruler *Structure* **18**, 1210-1219
4. Santoro, M. M., and Bolen, D. W. (1988) Unfolding free energy changes determined by the linear extrapolation method. 1. Unfolding of phenylmethanesulfonyl alpha-chymotrypsin using different denaturants *Biochemistry* **27**, 8063-8068
5. Moon, C. P., and Fleming, K. G. (2011) Side-chain hydrophobicity scale derived from transmembrane protein folding into lipid bilayers *Proc. Natl. Acad. Sci. U. S. A.* **108**, 10174-10177
6. The PyMOL Molecular Graphics System; Version 1.8 Schrödinger, L.
7. Wimley, W. C., and White, S. H. (1996) Experimentally determined hydrophobicity scale for proteins at membrane interfaces *Nat. Struct. Biol.* **3**, 842-848
8. Kyte, J., and Doolittle, R. F. (1982) A simple method for displaying the hydropathic character of a protein *J. Mol. Biol.* **157**, 105-132
9. Engelman, D. M., Steitz, T. A., and Goldman, A. (1986) Identifying nonpolar transbilayer helices in amino acid sequences of membrane proteins *Annu. Rev. Biophys. Biophys. Chem.* **15**, 321-353
10. Rose, G. D. (1978) Prediction of chain turns in globular proteins on a hydrophobic basis *Nature* **272**, 586-590
11. Hopp, T. P., and Woods, K. R. (1981) Prediction of protein antigenic determinants from amino acid sequences *Proc. Natl. Acad. Sci. U. S. A.* **78**, 3824-3828
12. Cornette, J. L., Cease, K. B., Margalit, H., Spouge, J. L., Berzofsky, J. A., and DeLisi, C. (1987) Hydrophobicity scales and computational techniques for detecting amphipathic structures in proteins *J. Mol. Biol.* **195**, 659-685
13. Eisenberg, D., Weiss, R. M., and Terwilliger, T. C. (1984) The hydrophobic moment detects periodicity in protein hydrophobicity *Proc. Natl. Acad. Sci. U. S. A.* **81**, 140-144
14. Janin, J. (1979) Surface and inside volumes in globular proteins *Nature* **277**, 491-492
15. Nozaki, Y., and Tanford, C. (1971) The solubility of amino acids and two glycine peptides in aqueous ethanol and dioxane solutions. Establishment of a hydrophobicity scale *J. Biol. Chem.* **246**, 2211-2217
16. Elazar, A., Weinstein, J., Biran, I., Fridman, Y., Bibi, E., and Fleishman, S. J. (2016) Mutational scanning reveals the determinants of protein insertion and association energetics in the plasma membrane *Elife* **5**
17. Wimley, W. C., Creamer, T. P., and White, S. H. (1996) Solvation energies of amino acid side chains and backbone in a family of host-guest pentapeptides *Biochemistry* **35**, 5109-5124
18. Hessa, T., Kim, H., Bihlmaier, K., Lundin, C., Boekel, J., Andersson, H., Nilsson, I., White, S. H., and von Heijne, G. (2005) Recognition of transmembrane helices by the endoplasmic reticulum translocon *Nature* **433**, 377-381

19. Guy, H. R. (1985) Amino acid side-chain partition energies and distribution of residues in soluble proteins *Biophys. J.* **47**, 61-70
20. Punta, M., and Maritan, A. (2003) A knowledge-based scale for amino acid membrane propensity *Proteins* **50**, 114-121
21. Senes, A., Chadi, D. C., Law, P. B., Walters, R. F., Nanda, V., and Degrado, W. F. (2007) E(z), a depth-dependent potential for assessing the energies of insertion of amino acid side-chains into membranes: derivation and applications to determining the orientation of transmembrane and interfacial helices *J. Mol. Biol.* **366**, 436-448
22. Koehler, J., Woetzel, N., Staritzbichler, R., Sanders, C. R., and Meiler, J. (2009) A unified hydrophobicity scale for multispan membrane proteins *Proteins* **76**, 13-29
23. Wolfenden, R., Andersson, L., Cullis, P. M., and Southgate, C. C. (1981) Affinities of amino acid side chains for solvent water *Biochemistry* **20**, 849-855
